# Emerging technologies and neuroscience-based approaches in dyslexia: a narrative review toward integrative and personalized solutions

**DOI:** 10.3389/fnhum.2025.1683924

**Published:** 2025-11-19

**Authors:** Rong Niu, Lu Ni, Feng Zhu

**Affiliations:** Hangzhou Women's Hospital (Hangzhou Maternity and Child Health Care Hospital), Hangzhou, Zhejiang, China

**Keywords:** developmental dyslexia, artificial intelligence, virtual reality, neurostimulation, deep learning, educational technology, neuroscience

## Abstract

**Background:**

Developmental dyslexia is a common neurodevelopmental disorder that impairs reading ability despite adequate intelligence and education, affecting up to 17% of children worldwide. Advances in neuroscience have revealed complex mechanisms involving phonological, visual, and temporal processing, with cross-linguistic variability. At the same time, technological innovation is driving a shift toward AI-powered diagnostics, immersive learning tools, and neurostimulation-based interventions.

**Methods:**

This narrative review synthesizes evidence from recent research published between 2015 and 2025, focusing on four thematic areas: (1) neurobiological underpinnings of dyslexia, (2) diagnostic innovations using AI and eye- or handwriting-based deep learning, (3) neurostimulation and immersive VR/AR interventions, and (4) policy, equity, and ethical considerations. Studies were identified through major academic databases and thematically analyzed to highlight trends, strengths, and limitations.

**Results:**

AI-based diagnostic tools using eye-tracking and handwriting features have achieved reported accuracies exceeding 80% in multiple pilot studies. VR/game-based programs and neurostimulation interventions (TMS, tDCS) have shown promising short-term effects on reading fluency and phonological processing, though evidence for long-term literacy transfer remains limited. Across studies, methodological heterogeneity and small sample sizes constrain generalizability. Significant disparities in access persist across socioeconomic, linguistic, and geographic contexts.

**Conclusions:**

While these technologies offer promising avenues for more personalized and scalable dyslexia care, their integration must be accompanied by stronger evidence, ethical safeguards, and equity-focused policies. Technology should augment, not replace, human interaction in inclusive education. Future research should prioritize larger trials, cross-linguistic validation, and sustainable implementation strategies.

## Introduction

Developmental dyslexia is considered one of the most prevalent yet complicated neurodevelopmental conditions that affect early and adult education worldwide. Dyslexia is a persistent disorder that impairs accurate and fluent word recognition, spelling, and decoding, despite adequate intelligence, educational opportunities, and instruction (Elliott and Grigorenko, 2024). Prevalence estimates vary according to language, assessment methods, and diagnostic criteria; nevertheless, international studies consistently indicate that approximately 5–17% of children are affected by dyslexia (Duranović et al., 2024). All these difficulties do not occur only in the classroom: dyslexia affects academic performance, mental health, self-confidence, career opportunities, and social acceptance, and it causes cascade effects on families, communities, and societies (Chalmpe and Vlachos, 2025).

## Classic theories to modern neuroscience

In the majority of cases, research based on phonological deficits has traditionally been conducted regarding dyslexia, and it has been suggested that the inability to manipulate the sounds of language (phonemes) at the linguistic level is the defining impairment in reading development (Gosiewska-Turek, 2025). This model was central to educational interventions for decades, as the idea of teaching phonics and remedial teaching became the focal point of the educational process (Tilanus et al., 2016). Nevertheless, it is clear that dyslexia is not a single disorder anymore. A complex syndrome has been identified with the help of advanced neuroimaging techniques (fMRI, EEG, DTI) and genetic research, in which visual processing, temporal (oscillatory) sampling, working memory, and executive function have been implicated (Banfi et al., 2019; Eden et al., 2016; Barbosa et al., 2019). Neurobiologically, it is evident that the development and functioning of a distributed network of brain regions, that is, the left superior temporal gyrus, inferior frontal gyrus, fusiform gyrus, and white matter pathways, are modified across alphabetic and logographic writing systems (Feng et al., 2020; Yu et al., 2020).

Importantly, cross-linguistic and cross-cultural observations show that the manifestations and severity of dyslexia depend on orthographic depth and, therefore, the structure of the language and the context of education (Elbeheri et al., 2024). For example, readers who have to learn to read using the opaque orthography of English face more of a decoding task than readers with a more transparent orthography system, such as Spanish or Finnish. More pronounced morphological and visual-orthographic abilities are also involved in logographic languages like Chinese, and the very idea of their neural dyslexia correlates (Pan et al., 2024). This evidence has led to a reconceptualization of the nature of dyslexia as a heterogeneous, neurodevelopmental disorder influenced by genetic, neurobiological, linguistic, and environmental processes (Mascheretti et al., 2017).

## The technology revolution: digital therapies, neurostimulation, and AI

The past few years have been characterized by revolutionary developments in the field of dyslexia study and treatment. With the introduction of artificial intelligence (AI), big data analytics, and digital technologies, it has become possible to develop new tools that help detect the disorder at its early stages, diagnose individuals, and employ evidence-based interventions. Diagnostic accuracies in machine learning models applied to studying handwriting, eye gaze, and speech now exceed 80% and are being implemented by translating them to other languages and populations (Gomolka et al., 2024; Liu et al., 2024). Remotely and digitally mediated interventions (VR, action video games, web/mobile apps, and neurofeedback interventions) have become more accessible, engaging, and saleable than ever before (Lorusso et al., 2024).

Meanwhile, non-invasive ways of influencing the brain based on neurostimulation, such as transcranial magnetic stimulation (TMS) and transcranial direct current stimulation (tDCS), can be used to modify neural activity within brain areas involved in reading. These methods have been shown to enhance reading abilities and help reading networks become more plastic (Rios et al., 2018; Turker et al., 2025). These developments are transforming the industry from its historical adaptation of a more general, “one-size-fits-all” remediation framework to a precision, neurobiologically informed framework of care that can be individually tailored to the profile and needs of individuals.

An international map or infographics with the estimated network of country/region-wide prevalence showing cross-linguistic variability and the magnitude of the problem (data sources: Pan et al., 2024; Seymour et al., 2003; Stein, 2018).

**Figure 1:**
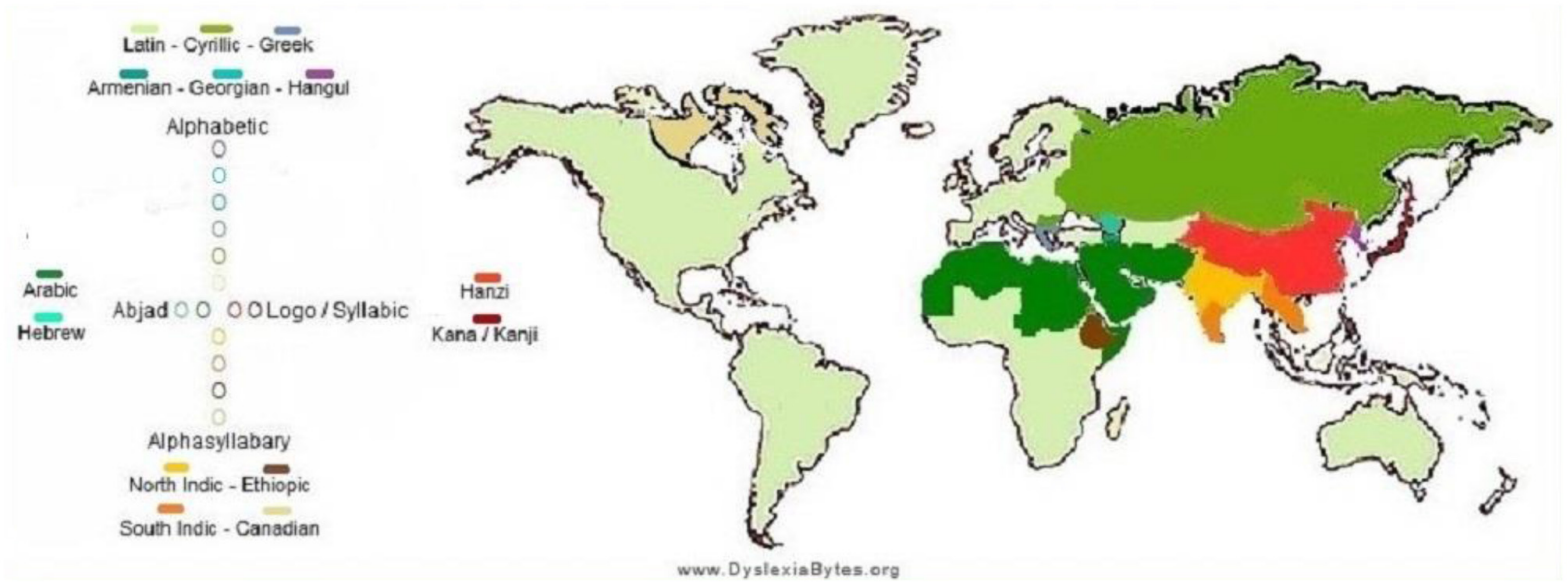
Global prevalence of dyslexia in children (source: https://hampsteadandfrognaltutors.org.uk/dyslexia-guide/).

Dyslexia prevalence data compiled from a global dataset (DyslexiaBytes) (Figure 1): Nigeria (20–33.3%), Saudi Arabia (14.2–30.6%), UK (4–15%), US (5–20%), Sweden (5–10%), and Russia (5–6%).

National prevalence estimates (e.g., US ~8.3%, UK ~5%; Poland, Italy, Singapore, the Czech Republic, and Norway) from KutestKids, DiscoveryABA, and WorldPopulationReview.

All the world frequencies of dyslexia are estimated widely, with a few as low as 2–5 percent in places like Singapore, Norway, Slovakia, and the Czech Republic, to as high as 30 percent in some areas, such as Nigeria and Saudi Arabia, among others. These fluctuations are associated with an intricate interaction between genetic, linguistic, and diagnostic components, as well as orthographic transparency and accessibility in terms of assessment.

*Online sources of data: DyslexiaBytes, Ambitions ABA Therapy, KutestKids*.

## The lack of resolution and a multidisciplinary, cross-traditional approach

Although these breakthroughs have been made, deeper issues continue to be impediments. Dyslexia is often weakly diagnosed and treated, particularly in disadvantaged contexts, and digital disparities exist along socioeconomic lines, language, and, to a lesser extent, access to technology (Fan et al., 2024). Not all subgroups respond equally well to many interventions due to additional barriers such as the presence of comorbid ADHD, poor socioeconomic status, or belonging to a linguistically diverse group (Yu et al., 2018). The deployment of AI and neurotechnology raises ethical concerns, such as privacy, potential bias in the technology, and the risk of overreliance on automated solutions (Salles and Farisco, 2024).

These issues call for an international, multidisciplinary, translational approach that links neuroscience, computer science, psychology, education, clinical medicine, public health, and policy. The combination of mechanistic insight, technology development and research, and implementation science promises to advance the field of dyslexia care to the personalized, scalable, and equitable care that individuals with dyslexia deserve.

## Purpose of this review

The purpose of this review is to explore the neural mechanisms and technology-based interventions in dyslexia and to highlight future synergistic, multidisciplinary, and ethical innovation in dyslexia research and practice:

- Cross-linguistic variability and neural mechanisms
- Early detection and diagnosis by means of AI and digital technology
- The effectiveness and operating mechanisms of digital and neurostimulation-based interventions
- Comparison of the results of classical and technology-assisted treatments
- Access, equity, and policy integration difficulties on a global level

Future synergistic, multidisciplinary, and ethical innovation: What follows is a discussion of directions in synergistic, multidisciplinary, and ethical innovation.

## Quality appraisal

Because this is a narrative review, formal appraisal tools such as GRADE or the Cochrane Risk of Bias framework were not applied. Instead, we implemented a critical appraisal process to ensure methodological transparency and minimize bias. Studies were assessed based on their relevance to the review themes, study design, sample size, and methodological clarity. Dual reviewer screening and consensus-based inclusion were used to enhance consistency and reduce subjectivity in study selection.

Because this is a narrative review, reported performance metrics (e.g., accuracy, sensitivity, specificity, AUC) are summarized as presented in each study. We did not conduct pooled statistical analyses or standardized benchmarking, as the included studies used different datasets, languages, participant age groups, and evaluation protocols. This variability is explicitly taken into account in our interpretation of findings.

## Current understanding of dyslexia neural mechanisms

**Figure 2:**
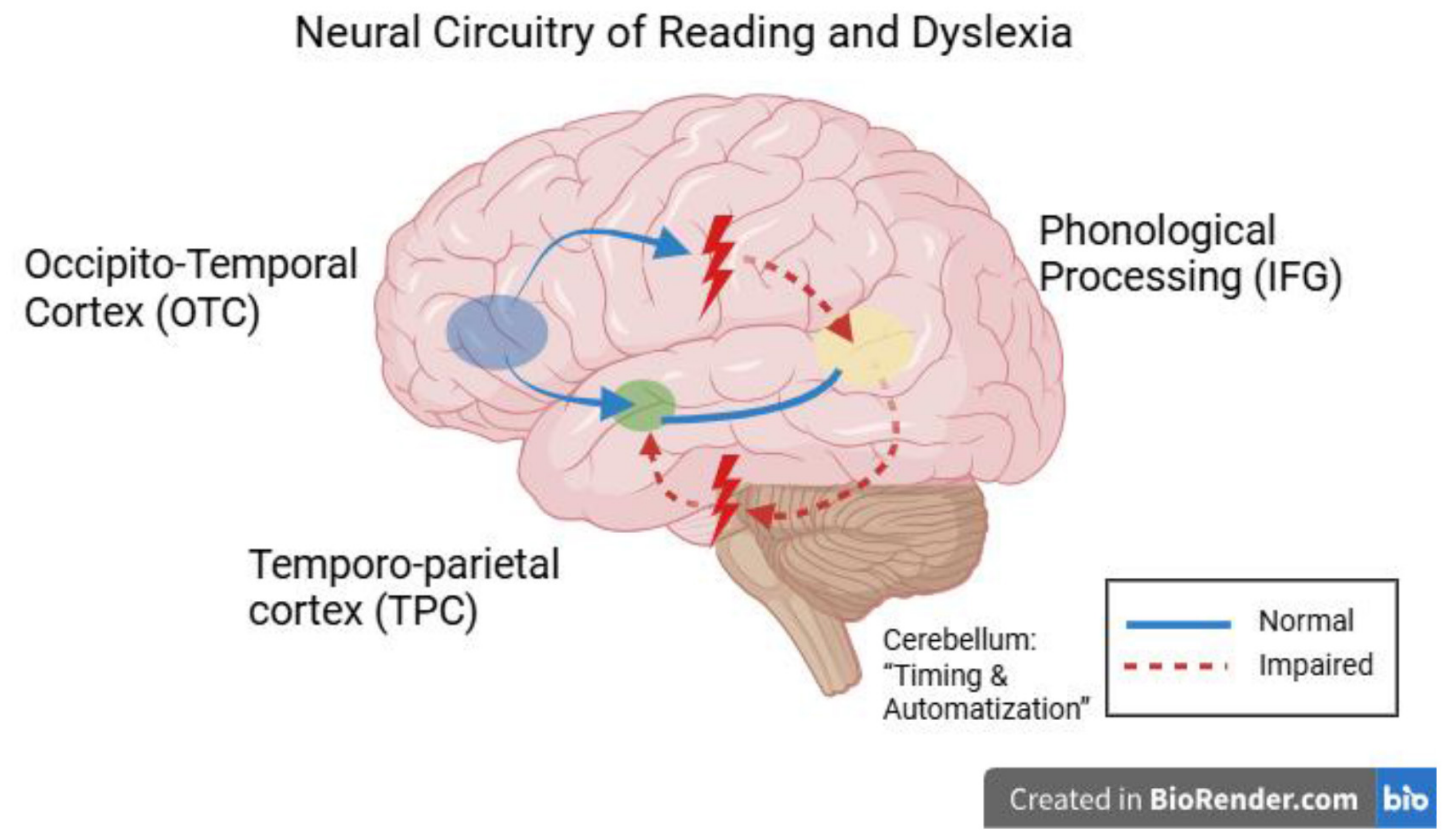
Figure of the brain on the left side of the head that are involved in reading and are routinely identified as unusual in children with dyslexia. The occipito-temporal cortex (OTC; visual word form area when decoding letters and words), temporo-parietal cortex (TPC; phonological decoding and print-to-sound mapping), and inferior frontal gyrus (IFG; word production and phonology). When we finally perform the reading task, those areas are hypoactive in dyslexic readers and present structural differences, including less gray matter in TPC and aberrant white matter connections. Dyslexia has also been linked to the cerebellum (not in the diagram), which may contribute to the impairment of control over when reading occurs and when reading automatizes, through its links with the frontal and parietal language regions (Hernández-Vásquez et al., 2023).

Developmental dyslexia represents a neurodevelopmental reading disorder in which acquiring reading skills is unexpectedly difficult, even though there is no deficiency in intelligence or education. Studies over the decades have focused on a cluster of brain areas that are abnormally developed and influence the phonological and reading disorders of dyslexic children. The left part of the brain, including the temporo-parietal cortex (TPC), occipito-temporal cortex (OTC), and inferior frontal cortex (IFG), in normal readers, is said to perform in a coordinated effort to map print to sound and meaning. The structure and functioning of these regions are, however, modified in dyslexic children and are accompanied by abnormalities in brain oscillation and connectivity, pointing toward a multifactorial implication of the disorder at the neural level. Evidence from early neuroimaging studies and EEG suggests that many of these neural disparities are also present prior to reading instruction (in infants and pre-readers at risk of developing dyslexia). Prior to the list, we discuss five dominant and proposed neural dyslexia mechanisms in children—phonological deficits, impaired visual processing, anomalous temporal processing (oscillatory explanation), disrupted neural connectivity, and cross-linguistic and demographic variability—using recent evidence (2020–2025) with particular focus on neuroimaging results (fMRI, EEG/MEG, DTI) and rigorous peer-reviewed results (Figure 2).

## Phonological deficits

**Figure 3:**
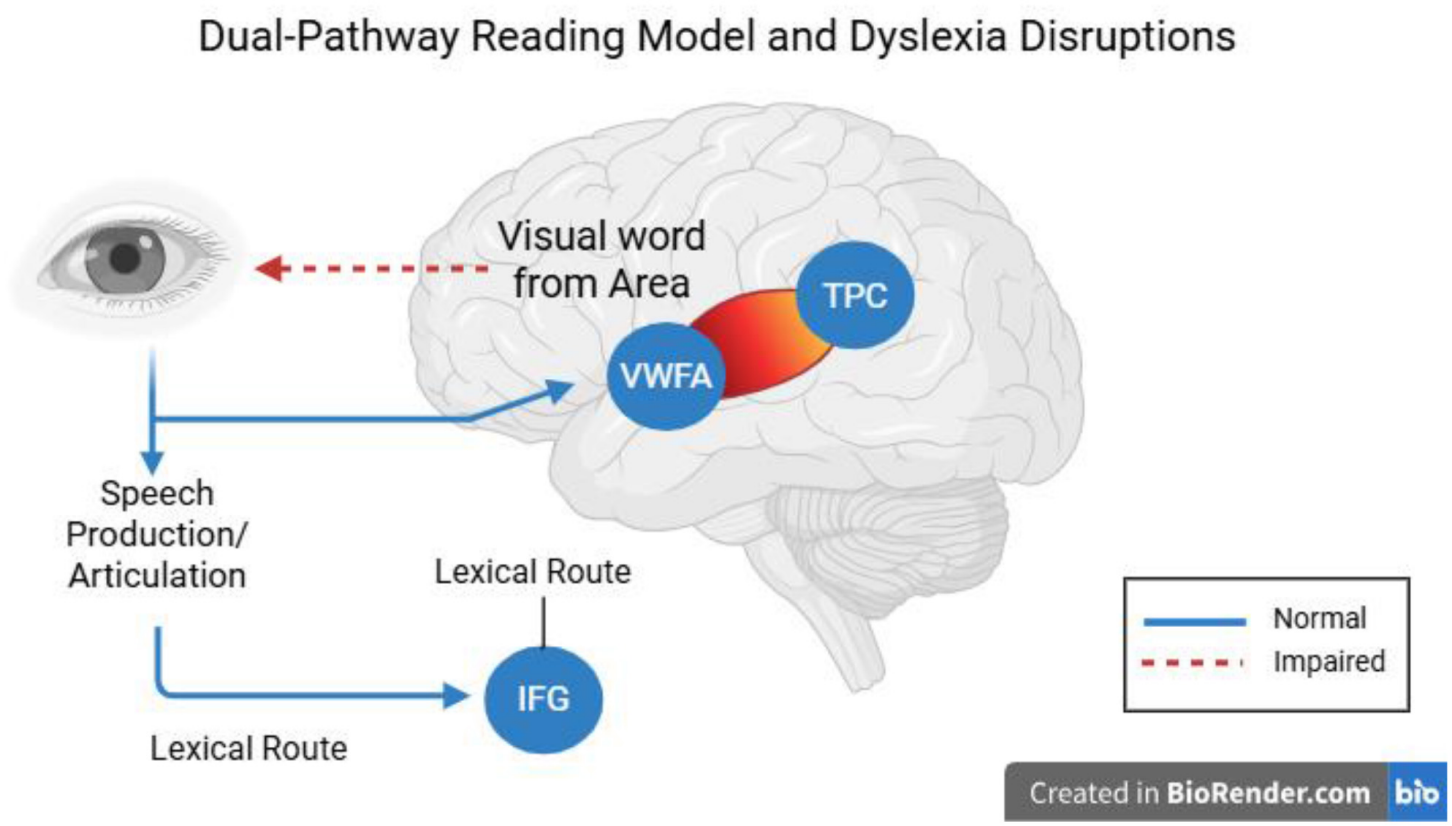
Dual-pathway reading model showing disrupted activation in the temporo-parietal cortex (TPC) and its impact on phonological decoding in dyslexia.

A phonological deficit hypothesis of dyslexia is the idea that dyslexics have a central inability to represent and manipulate speech sounds (phonemes). As shown in Figure 3, the dual-pathway reading model highlights how disrupted activity in the temporo-parietal cortex affects phonological decoding, providing a neural basis for these behavioral deficits. Behavioral evidence (children with dyslexia have trouble with phonological awareness, rapid naming, and repetition of non-words) strongly supports this theory, and there are obvious neural correlates. The consistent finding in functional neuroimaging is that children with phonological deficits underactivate left hemisphere language regions when performing phonological tasks (Richlan, 2020; Hernández-Vásquez et al., 2023). Specifically, underactivation of the left posterior temporo-parietal cortex (TPC)—regions surrounding the supramarginal and angular gyri and posterior superior temporal gyrus (Wernicke's area)—occurs during tasks where dyslexic readers are attempting to map letters onto sounds or deal with phonology. Left TPC hypoactivation during phonological processing tasks is a reliable pattern observed in both children and adults with dyslexia (Hernández-Vásquez et al., 2023). In normal readers, adding to the requirements of a phonological decoding task (e.g., reading pseudowords) would increase TPC activation, whereas dyslexic readers do not display this normal tendency to increase. This indicates that there is functional impairment in the neural graph-to-phoneme transformation. Likewise, meta-analyses across studies have found the left superior temporal gyrus (STG)—another major auditory-phonological system involved in dyslexia—to be a convergence site of abnormalities in dyslexia. A multimodal MRI meta-analysis comparing dyslexic readers across alphabetic and logographic languages revealed structural and functional overlap in abnormalities of the left STG, indicating a common neural signature of the phonological deficit (Yan et al., 2021). Additionally, there is decreased volume of gray matter and decreased fMRI activation in regions of the left STG/TPC when dyslexic children, compared to controls, perform phonological tasks (e.g., judging rhymes or listening to phonemes). The results can be framed by the opinion that the defective phonological cortex should inhibit the formation of effective reading networks (Figure 3).

The case is also reinforced by the anatomical study in relation to the phonological basis of dyslexia. Research with post-mortem and structural MRI (including classic yet historically significant studies by Galaburda) indicated micro-architectural abnormalities (ectopias, abnormalities of neuronal migration) in the left perisylvian language areas of dyslexics that potentially precondition phonological processing problems. Reduced gray matter volume in the left temporo-parietal regions has been confirmed using recent structural MRI, as observed in dyslexic children. Table 1 summarizes key neuroimaging studies that consistently report temporo-parietal hypoactivation and its link to phonological deficits (Liu et al., 2022). Diffusion tensor imaging (DTI) has also demonstrated impaired white matter pathways that link phonological language areas. For example, reduced fractional anisotropy (FA), together with disarrayed structure, is very common in the left arcuate fasciculus (the Big Dorsal pathway that is thought to interconnect posterior temporal language regions with the frontal lobe) and is commonly reduced in children with dyslexia, especially regarding phonological awareness and decoding scores. According to longitudinal studies, the existence of such differences in white matter precedes literacy training; in fact, inferior left arcuate fasciculus connectivity develops atypically in infants and pre-readers with a familial risk of dyslexia (Rezaei et al., 2024). This indicates that a large proportion of dyslexic children are born with less effective phonological wiring in the brain.

**Table 1:** Summary of key neuroimaging evidence supporting temporo-parietal hypoactivation during phonological processing tasks in dyslexic readers.

**Study**	**Sample and method**	**Key findings on phonological deficits**
Hernández-Vásquez et al. (2023)	Review of fMRI/EEG studies (2000s−2020s); children and adults with dyslexia	Hypoactivation of the left temporo-parietal cortex (TPC) under maximum phonological demands; intra-subject failure to activate the left TPC, as phonological demands increase in dyslexics with higher phonological demands. Likewise, shows abnormal activation of the left OTC and IFG and smaller gray matter in the left TPC.
Yan et al. (2021)	Meta-analysis of 40 fMRI/VBM studies across languages (alphabetic and Chinese)	Presence of a convergent left superior temporal gyrus (STG) deficit among all languages in dyslexics suggesting there is a phonological-processing impairment in dyslexia. Noted also differences with language (e.g., Chinese dyslexics given more activity in IFG).
Vandermosten et al. (2012)	DTI in children (pre-readers through readers)	The phoneme awareness and the speech sound processing are linked to the integrity of the left arcuate fasciculus. A phonological pathway deficit was also evidenced by dyslexic children responding with decreased connectivity in the left arcuate.
Marchesotti et al. (2020)	EEG and intervention in dyslexic adults (extended to children in theory)	Left auditory cortex 30 Hz brain stimulation (tACS) transiently enhanced phonemic decoding by normalizing phonological deficit-related 30 Hz (low-gamma) auditory oscillations in a total of 5 dyslexic participants.

The inferior frontal gyrus (IFG), known to play a role in phonological rehearsal and articulation, shows notable mixed results. There have been reports of hyperactivation of the left IFG (Broca's area) during phonological tasks in dyslexic children (Corral Rosado, 2024), which has been proposed to reflect an attempt at compensation (i.e., ineffective recruitment of frontal regions by dyslexic readers to support phonological analysis) (Newsham, 2022). In fact, it has been found that normal readers exhibit an age-related decline in their IFG activation recruitment during reading, whereas dyslexic readers exhibit an age-related increase in IFG activation. Perhaps as reading becomes increasingly automatic, their reading is more plausibly evidence of effortful phonological decoding (Yan et al., 2024). However, other studies note IFG hypoactivation or no difference, indicating some variability in the compensation of phonological deficits in the brains of children. Regardless of such caveats, the general evidence suggests that one definitive ingredient of dyslexia is a deficiency in the phonological loop, specifically the underdevelopment of the left temporo-parietal area and fragile fronto-temporal connections (Abbott and Love, 2023). This brain configuration corresponds with the findings of the phonological theory of dyslexia, which proposes that dyslexic children face difficulty in handling the sound structure of language, and in turn, this difficulty affects their ability to learn the matching of letters and sounds (Table 1).

## Visual processing

Besides the existence of phonological problems, most dyslexic children have visual processing impairments that influence reading (Mantovani et al., 2021). These include issues with processing swift visual information, aberrant perception of movement, poor visual memory of letters, and a low span of visual attention (the amount of letters/symbols one can scan accurately within his or her vision at a glance). There are two major (and not mutually exclusive) theories that address the visual deficits in dyslexia: the magnocellular-dorsal pathway theory and the deficit in visual attention span theory. The latest studies, on the other hand, have shown that a small group of dyslexic children do have neural impairments in visual pathways, which may run parallel with phonological impairments in reading (Leung et al., 2022). A competing theory, the magnocellular-dorsal theory, posits a dysfunction in the magnocellular neurons of the visual system that process low-contrast and fast, high-resolution visual stimuli and motion, which feed into the dorsal visual stream (Stein, 2018; Wilkins and Evans, 2024). Psychophysical and neurological evidence can be used to support this theory: for example, dyslexics tend to exhibit higher thresholds in lateral motion detection as well as flicker detection (Ji and Bi, 2020), and post-mortem tests of dyslexics have revealed irregularities in the magnocellular layers of the lateral geniculate nucleus (LGN). A general body of research supports the idea that in dyslexia, there is gross evidence of retarded development of magnocellular neurons (Kristjánsson and Sigurdardottir, 2023) that affect the timing of visual processing, as cited in Ji and Bi (2020) studies confirming that Chinese children with dyslexia perform poorly on tasks that involve rapid visual processing of motion/form, particularly in high visual noise conditions. These findings imply that dyslexics have difficulty with signal-in-noise extraction in dynamic visual displays, consistent with a deficit in the magnocellular pathway or associated noise exclusion disability. Neuroimaging findings also indicate lower activity in the dorsal visual stream (such as V5/MT, which supports the detection of motion) and poor blood-oxygenated level of dissimilarity between the occipital (visual regions) and temporal cortex in readers with dyslexia during reading exercises (Hernández-Vásquez et al., 2023). Dyslexic children tend to have low EEG connectivity between the occipital and inferior-temporal regions, a pathway involved in the analysis of visual letters regardless of its connection to the word form area (Hernández-Vásquez et al., 2023). These may be evidence of an immature magnocellular-dorsal pathway to shuttle visual information quickly to the language system. Table 2 presents a summary of key studies on visual processing deficits, showing consistent evidence of dorsal stream dysfunction and reduced visual attention span across different languages.

**Table 2:** Visual processing impairments in dyslexia: selected findings.

**Study**	**Focus**	**Key findings**
Ji and Bi (2020)	Magnocellular vs. Noise-Exclusion in Chinese dyslexic children (behavioral study)	Motion and form sensitivity scores for dyslexic children were poor relative to control children only in the high visual noise, not low noise conditions. Suggests that visual dysfunction in dyslexia is associated with downward filtering difficulty (engaging a noise-exclusion deficit), whereas basic magnocellular performance was less poor in no-noise conditions.
Zhao et al. (2023)	White matter connectivity sub-networks (French dyslexic children)	Found a different set of neural circuits regarding visual attention span compared to phonological weaknesses. Less efficient structural connectivity within a left occipito-parietal network was linked to less effective visual–attentional processing (VAS), but phonological weaknesses were associated with connectivity within left temporo-parietal and left frontal networks. Sustains the fact of visual-attentional form of dyslexia.
Hernández-Vásquez et al. (2023)	fMRI/EEG review of dyslexia neural correlates	Hypoactivation of the left occipito-temporal cortex (VWFA) during word reading is a strong neural indicator in children with dyslexia. Associated with impairments in rapid naming and word recognition. Indicates that there is a failure in the normal development of the visual word form system. Is also associated with diminished occipital-temporal EEG connectivity, which indicates impaired flow of information at the visual-to-lexical stage.
Stein (2018)	Review of magnocellular theory evidence	There is substantial evidence compiled of defective development of magnocellular neurons in dyslexia affecting the dorsal visual stream. Abnormal magnocellular circuitry dysfunction results in timing impairments in visual sequencing (e.g., motion detection deficits/stable binocular fixation) which could contribute to reading disturbances, though interventions for it are still in development.

Visual deficits may be explained by the noise exclusion theory, which emphasizes the inability to filter irrelevant visual noise (Sperling et al., 2005). Interestingly, a study identified that performance deficits on motion and contrast tasks in Chinese dyslexic children occurred mainly in high-noise rather than low-noise displays, indicating that the deficiency is a noise-exclusion deficit rather than a pure magnocellular deficit (Ji and Bi, 2020). This is a controversy not yet fully deciphered, but in both explanations, there is atypical neural processing of early visual circuits involved. Regardless of unstable magnocellular responses or poor noise suppression, dyslexic brains are less efficient in processing speedy visual inputs, which might disrupt the visual guidance of reading (e.g., control of eye movements, sequential processing of the letters).

In addition to having poor low-level vision, children with dyslexia also have a weak visual attention span (VAS) ability, which is their ability to process many letters simultaneously or visuospatial items. This idea, which has its origins in the cognitive works of Valdois and others, is found to be supported by the brain. The study supports the existence of a dissociation between the neural circuits underlying VAS and phonological deficits in dyslexia (Zhao et al., 2023). Based on white-matter network analysis in French dyslexic children, it was found that connectivity in one of the critical networks (occipito-parietal, including the left superior occipital gyrus and the associated dorsal cortex) determined the severity of VAS deficits in the children independently of phonology. In contrast, the phonological deficits were associated with connectivity anomalies in a left temporo-parieto-occipital system (centered around the left middle temporal gyrus hub) and a frontal system. These observations indicate that the primary impairment of some children with dyslexia is the amount of visual processing that can occur in parallel (associated with connections in the dorsal visual stream), as opposed to traditional phonological coding. A small visual attention span might complicate reading by decreasing the quantity of letters that are read at any eye fixation, hence delaying word recognition. Neuropsychologically, this is matched with a subtype of dyslexia in which a child might do quite well on phonological tasks but still not read fluently, owing to visual-attentional limits (masquerading as what might be termed attentional dyslexia).

More importantly, an anomalous activation of the occipito-temporal cortex (OTC) constitutes a well-replicated neural indicator of dyslexia. This is illustrated in Figure 2, which depicts the brain regions typically affected in dyslexic readers, including the left fusiform gyrus (VWFA) and its disrupted connectivity with phonological areas. The left fusiform gyrus (VWFA) is typically sensitive to print in literate adults, so that other familiar strings of letters consistently cause recruitment of the area (Danelli et al., 2017). Assessment of lifting the finger is blunted in dyslexic readers. Functional MRI displays reliably decreased activity in the left OTC/VWFA in dyslexic children when reading words or pseudowords. This underactivation has been found in the early stages of development and persists into adulthood, signifying the inability to effectively utilize and develop this area for smooth recognition of words. This aberrant OTC activation occurs persistently in individuals with dyslexia, as it is correlated with poor letter and word recognition and slow rapid automatized naming (RAN) (Hernández-Vásquez et al., 2023). Structurally, there are also reports of decreased gray matter or cortical thickness surrounding the VWFA in dyslexic readers (though findings are mixed). The causal nature is controversial: VWFA underfunctioning might be the result of lower print exposure or possibly a genetic predisposition. Longitudinal studies in at-risk pre-readers imply a certain level of pre-existing disparity: before instruction in reading, at-risk children demonstrate reduced print-specific brain reactions within occipito-temporal areas and irregular connections of these regions to language areas (Sinclair-Amend, 2022). Table 2 summarizes the main AI-based diagnostic approaches for dyslexia. As shown, deep learning models applied to handwriting and eye-tracking data demonstrate consistently high classification accuracy, often exceeding 80%, across multiple studies and languages. In total, the data suggest that dyslexia is commonly characterized by a 2-fold impairment: a main phonological impairment and a second orthographic/visual word form impairment, with some children showing one or the other. In many children, visual processing anomalies, such as subnormal magnocellular motion detectors, an abbreviated visual attention span, and poor use of the VWFA, therefore, constitute an essential component of the neurobiological view of dyslexia (Table 2).

## Temporal processing and oscillations

A second line of inquiry has pointed to deficits in temporal processing, i.e., the timing of sensory and perceptual mechanisms, as a factor in dyslexia. It is worth noting that since 2010, abnormal brain oscillations in dyslexic brains have been a point of interest with the so-called temporal sampling framework (TSF) proposed by Goswami et al. The TSF hypothesizes that dyslexia involves a problem of phase-locking brain oscillatory rhythms to the temporal pattern of speech, resulting in an inefficient sampling of speech information at several time scales (slow: the syllabic and prosodic cues; fast: the phonemic cues). This opinion is supported by EEG/MEG experiments in recent years (2020–2025), where dyslexic children are compared with typical readers and demonstrate a difference in oscillatory power and synchronization in frequency bands (O'Brien and Yeatman, 2021).

Electrophysiological signatures: Using resting-state and task EEG, children with dyslexia were generally found to exhibit greater low-frequency power (delta 0.54 Hz and theta 48 Hz) and reduced to mid-frequency power (alpha ~812 Hz) in brain areas supporting reading. As an example, dyslexic people have a significantly abnormal increase in delta/theta activity in the frontal, temporal, and parietal cortex compared to controls, together with an abnormal decrease in the normally prevalent alpha rhythm (Taskov and Dushanova, 2022). The abnormalities are mostly confined to the left inferior parietal areas of the reading network that have been referred to as the decoding areas of importance. Delta and theta bands are related to the speech rate of prosodic and syllabic information, respectively (Boissonneau et al., 2022). A loss of theta oscillations (~5 Hz) that takes place in dyslexic children is thought to interfere with the brain's encoding of syllable-sized chunks of sound, since theta oscillations naturally go in step with syllable timing. Failure of the brain to consistently synchronize to syllabic units has the potential to impede the development of consistent phonological representations and multi-sensory (audio-visual) integration required in reading. Attenuation of alpha power in dyslexia is also linked with phonological problems in some studies because alpha-band activity is believed to control information-gating and attention. The overall result is that the dyslexic brain's oscillation environment can be said to be out of phase with the temporal rhythm of speech and print, leading to poor sampling of the input (Hatfield, 2023).

Neural synchronization to speech: A major historical thrust of studies on dyslexia has been to investigate brain entrainment to variations in spoken language. There is a characteristic weaker phase-locking of neural activity to amplitude modulations in speech by dyslexic children across the low-frequency (delta/theta) range that time syllable duration and prosodic stress. For example, they display a less consistent activation of the auditory cortex to rhythmic sequences of syllables or in response to the embedded prosody of sentences, compared to typical readers (Power et al., 2016; Soltész et al., 2013). Another intervention study showed that improving phonemic discrimination in individuals with dyslexia can enhance neural rhythm (Marchesotti et al., 2020). As mentioned above, the impairment in the 30-Hz (gamma) oscillations of the left auditory cortex in dyslexic adults was detected, representing the window of temporal integration of phonemes (~25 40 ms) (Marchesotti et al., 2020). They could acutely modulate phonological performance by externally creating the hypothetical same 30-Hz rhythm. This may imply that the brains of dyslexics do not spontaneously produce sufficiently robust gamma oscillations to analyze sudden changes in acoustics (such as consonant shifts), which is a primary feature of the temporal processing deficit. In fact, there is an emerging idea that dyslexia is a multi-timescale temporal disorder: slower oscillations (delta/theta) due to syllables are mistimed, and faster oscillations (beta/gamma) to phonemes are weaker or at unfavorable frequencies (Rufener and Zaehle, 2021). In line with this, weaker theta and gamma synchronization in response to speech sounds was found in dyslexic readers, and this finding corresponds to low phonological awareness (Archer et al., 2020).

Neural noise hypothesis: The notion of “neural noise” also relates to oscillation timing. The noisier neural processing response may be apparent in dyslexic brains and cause a lower signal-to-noise ratio in the perception of the timing of input (Turri et al., 2023). In a 2021 eLife study, the theory was re-tested by analyzing EEG variability (trial-to-trial variability) in the neural responses of dyslexics and was partly supported by increased trial-to-trial variability (dyslexics) and oscillatory typical differences (Daniel and Dinstein, 2021). This raised level of cortical noise would impair steady-phase locking to stimuli. However, there is inconclusive evidence; some studies have concluded that the brains of dyslexics need not be overall noisier but fail at the level of matching oscillations with stimuli.

Oscillatory connectivity: Along with local oscillatory power, dyslexia can also relate to disturbances in the synchronization of brain regions. Variants of EEG coherence and phase synchronization demonstrate that the usual decreased long-range connectivity of the reading network between disparate cortical locations in the theta band is seen in dyslexic children (Formoso et al., 2021). For example, poor interconnections at theta frequencies have been reported between the left and right superior temporal gyrus, left occipital (visual), and inferior-temporal regions, as well as other reading-related domains in dyslexic children (Hernández-Vásquez et al., 2023). This suggests that auditory, visual, and language areas have neural assemblies that are less synchronized in time, which may hinder their ability to integrate sounds and letters correctly. A worldwide network overview in this review reported that dyslexics also exhibited a pattern of deficiency in the spectrum of the beta band (15–30 Hz), another rhythm associated with cognitive processing. Interestingly, compensatory patterns are also observed: in severely dysfluent dyslexic children, greater connectivity was found between left central (motor) regions and right inferior-temporal and occipital regions during a visual word task. This may reflect a different route that has been recruited (e.g., activating the right hemisphere visual areas in support of word recognition). In summary, such results can be linked to the temporal processing account and the connectivity account, where the inability of the networks to synchronize well—particularly in low frequencies that facilitate speech parsing (theta) and higher frequencies that parse phonemic detail (gamma)—is often related to dyslexia.

It should also be mentioned that auditory temporal processing impairments have been reported over many years as behavioral abnormalities in dyslexia (e.g., Tallal's theory of rapid auditory processing) (Tallal et al., 1993). Dyslexic children tend to have problems distinguishing bouts of rapid tones or recognizing short pauses of sound, which suggests slow auditory timing. The modern oscillation study furnishes a neurophysiological background to these findings: unless the brain's natural oscillators are flocking to fast events, the child will not manage to perceive transient or quickly succeeded sounds, which is the background to phonemic confusion. The combined evidence itself is overwhelming, leading to the conclusion that dyslexia is partially a neural timing disorder over the 2020–2025 time period. Anomalous brain rhythms and timekeeping issues (particularly regarding the way that the brain synchronizes to the rhythms of spoken language) play a significant role in the phonological and sensory dyslexia symptoms (Parvez et al., 2024).

## Neural connectivity

Dyslexia may not only be considered as focal regional impairments but also as a disconnection syndrome of networks sustaining reading. The connectivity of the brain, in the form of neural pathways (structural) and networks (functional), is therefore important in ascertaining the visual, auditory, and language processes involved in reading. An emerging literature of diffusion MRI and functional connectivity findings (both with and without the use of DTI, tractography, and resting-state fMRI) suggests that dyslexic children are abnormally connected in the reading circuit.

White matter anomalies: Deficits in the left hemisphere network have been shown consistently in the major language pathways of the white matter in dyslexia. The most commonly reported tract involves the left arcuate fasciculus (AF) of the superior longitudinal fasciculus, which links posterior regions (e.g., Wernicke) to anterior regions in language (e.g., Broca). Dyslexic children probably exhibit lower levels of fractional anisotropy and volume of the left AF, which indicates poorly organized or denser bundling of nerve fibers. The phonological deficits identified above are probably a result of this structural disconnection because the AF conveys phonological information to map sound to speech production. For example, FA in the left AF has been shown to predict phoneme awareness skills, with dyslexic pre-readers exhibiting significantly weaker FA in this tract compared to typical pre-readers (Vandermosten et al., 2012). Other involved tracts besides the AF comprise the left inferior longitudinal fasciculus (ILF) and the inferior fronto-occipital fasciculus (IFOF), which integrate visual word form areas with frontal and anterior language and semantic hubs. Such ventral stream pathways are not always as coherent in dyslexia, which correlates with orthographic processing problems (e.g., poor sight word reading efficiency). Such a conclusion was made in a recent systematic review of the evidence base of DTI research (2021), which states that the pattern of atypical white matter connections is a consistent finding in developmental dyslexia, particularly left-hemisphere-based reading paths (Thakkar, 2021). Most remarkably, most of these differences can be found early in the lives of the children. Findings suggest a disconnection in children who later developed dyslexia, characterized by less well-organized white matter in the tracts of the left temporo-parietal region at the age of five, before they had received formal reading instruction (Kuhl et al., 2020). The structural deficits are not global brain abnormalities but rather pertain to the networks of reading and language.

Functional connectivity: The inter-regional coordination can also be different in dyslexic individuals, although they employ similar regions during a task. This impaired intrinsic connectivity in the reading network has been observed using resting-state fMRI and is evident in dyslexic children (Martins et al., 2025). For example, underconnectivity between the left fusiform gyrus (VWFA) and the temporo-parietal cortex has been reported in dyslexic children aged 8–12 years, indicating reduced coupling with visual and phonological regions even in the absence of a task (Cheema et al., 2021; Meri et al., 2020). Associative connections also increase in task-based connectivity analysis, where dyslexics are not as effective in linking regions when reading. Irregular inter-hemispheric connections have also been reported: fMRI found that dyslexic children have increased connectivity between the left and right IFG (possibly as a compensation mechanism in the right hemisphere) and decreased connectivity between the left IFG and the left temporo-parietal cortex when compared with controls (Richards and Berninger, 2008). This suggests that there might be rerouting of the activity due to alternative networks.

A functional disconnection, related to the electrophysiological findings in the connectivity described in the previous section (a decreased coherence within the theta band between dissimilar cortical regions), is represented by the findings. To conclude, dyslexia involves the disintegration of the orderly coordination of networks necessary to read smoothly: the visual, phonological, and higher language networks do not talk to each other at the right time or with the maximum strength. These results are clinically relevant; they could be interpreted as reasons why multisensory training (which seeks to orthogonally activate visual letters and auditory sounds, e.g., simultaneously) can, on occasion, remediate reading (this binds visual letters and auditory sounds much more closely together). Particularly strong evidence in support of connectivity-based identification of dyslexia concerns infants and pre-readers. The integrity of the left AF in 5-year-old pre-readers has been shown to predict their later reading abilities. More recent research indicates that infants at risk for dyslexia exhibit abnormal structure of the left arcuate fasciculus, and that pre-readers with familial risk already show functional and structural disturbances in left hemisphere reading regions, including the OTC and TPC (Saygin et al., 2013; Beach et al., 2022). This implies that poor connectivity is not a consequence of reading failure but probably a causal factor: a child who, depending on genetic or developmental considerations, has a less connected reading circuitry will not fare as well once subjected to formal reading instruction. The hope is that with early detection of such connectivity problems (through infant neuroimaging or early screening EEGs), interventions can be used to reinforce those neural connections (early intervention has, in fact, been shown to lead to normalization of some connectivity measures).

**Figure 4:**
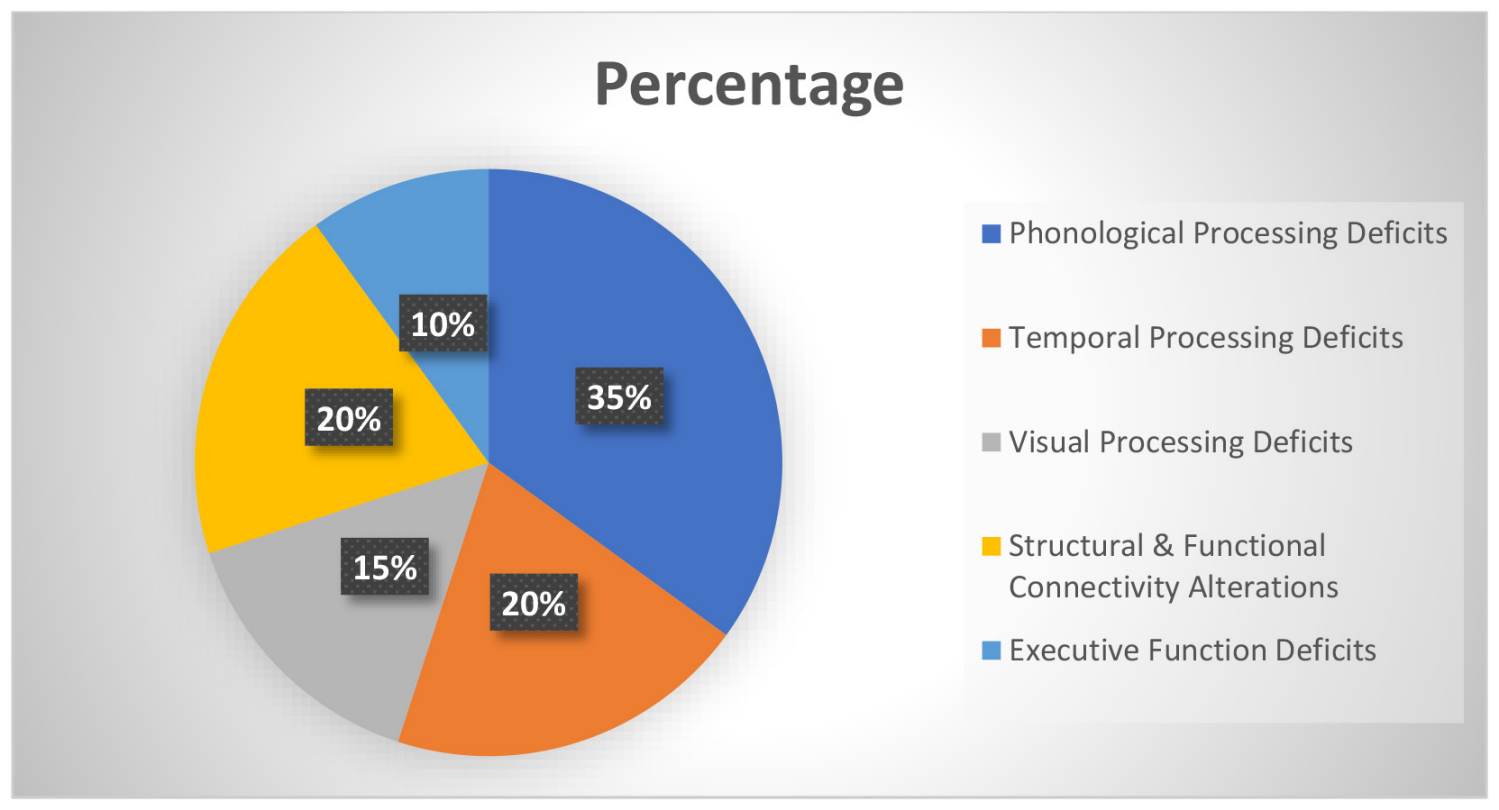
Estimated distribution of the primary neural deficits contributing to dyslexia.

Brain imaging of connectivity: The reduced white matter connectivity between the reading networks in both orthographic and phonological dysfunctions has been attributed to dyslexia (Sihvonen et al., 2021). Conversely, previous reports have revealed that effective reading intervention may result in an increase of white matter fractional anisotropy (FA) and enhanced connections in corresponding tracts. This has first been demonstrated in adults (Keller and Just, 2009), and delays in neuroplasticity have now also been found in children: in dyslexic children, after intensive phonological training, not only are reading scores improved, but connectivity in left hemisphere fiber bundles also increases, and activation between the VWFA and language areas becomes more synchronized. Such plasticity is evidence that connectomics is a dynamic feature and a remediable target (Figure 4).

## Cross-linguistic and demographic factors

Dyslexia has been described as neurobiologically universal in that reading engages the same broad network across various languages. Nevertheless, research from cross-linguistic studies has indicated that the effects of the neural deficits associated with dyslexia may differ based on the writing system and language properties (including writing depth, script, and phonological system). In addition, there is increasing evidence that other demographic variables, such as age, sex, and bilingualism, can also modulate the dyslexic neural profile, although these factors are less well-investigated compared to language. Here we synthesize results from cross-language and cross-population comparisons of the neural mechanisms of dyslexia.

A seminal cross-language fMRI study demonstrated that, despite distinct behavioral profiles, left temporo-parietal hypoactivation was common among Italian, French, and English dyslexics, indicating a consistent phonological deficit that interferes with their language-specific performance (Paulesu et al., 2001). Building on this foundation, current meta-reviews validate a framework of cross-modal abnormalities in dyslexia, in addition to language-specific variations (Richlan, 2020). A systematic review of dyslexia found core areas of the left hemisphere reading network—composed of the occipito-temporal, temporo-parietal, and inferior frontal cortex—underactivated among dyslexic readers across all linguistic systems (Figure 2). This aligns with the fact that, regardless of script (alphabetic, syllabic, or logographic), dyslexics have difficulty integrating print into sound, as evidenced by the underactivation of the left VWFA/OTC, left STG/TPC, and, in some cases, left IFG. Richlan's meta-analysis observed that the differences among languages primarily consist of the extent and intensity of these activations. For example, shallow orthographies (such as Italian or German, where there is a near one-to-one grapheme-phoneme correspondence) are associated with reduced hypoactivation in phonological regions compared to deep orthographies (such as English) since, in shallow orthographies, reading relies less on intensive phonological decoding after learning the fundamentals of decoding. Accordingly, Italian dyslexics are primarily slow readers (slow fluency), exhibiting manifestations of weaker decoding, and scans of their brains show less intense underactivation in the temporo-parietal areas, although it is still present.

Alphabetic vs. logographic: One very striking difference is between alphabetic writing (English, French, etc.) and logographic or morpho-syllabic writing such as that of Chinese. Alphabetic reading learning to read is based on the mapping of letters to phonemes; reading Chinese characters maps onto syllables and morphemes, requiring increasingly visuospatial and other memory processing loads. A multimodal neuroimaging meta-analysis, one that compared dyslexia among morpho-syllabic languages (e.g., Chinese) and an alphabetic language, was also conducted (Share, 2025). They discovered that though both shared left STG disruption (the aforementioned universal phonological locus), other regions exhibited divergent patterns: Chinese dyslexics had much more severe decreases in activation and gray matter volume in the dorsal (inferior frontal) part of the left IFG and significantly deeper reductions than alphabetic dyslexics in the left middle temporal gyrus and fusiform (occipito-temporal) gyrus; alphabetic-language dyslexics exhibited a much more severe reduction in gray matter volume in the middle temporal gyrus and fusiform gyrus. That is to say, Chinese dyslexia seems to preferentially challenge the frontal articulatory and, perhaps, tonal processing systems (because Chinese reading involves mapping to the spoken syllable and may recruit working memory and tone subsystems in IFG), and has relatively weaker deficits in the orthographic/visual word form area (fusiform gyrus) and in semantic-related middle temporal regions. In a meta-analysis of Chinese vs. alphabetic dyslexic children, similar shared and different abnormalities were reported (Li and Bi, 2022), with shared left-temporo-parietal underactivity, Chinese peculiar under-recruitment of frontal regions, and alphabetic peculiar under-recruitment of visual regions (accounting for the demands of scripts). Such differences are obvious: unlike alphabetic characters, Chinese characters are more visually complex and less systematically phonetic in their structure, and memorization of visual orthography plays a more important role (visuo-spatial working memory is frequently impaired in dyslexic Chinese readers), whereas the conversion of graphemes to phonemes is problematic in alphabetic dyslexics, with strong fusiform and temporo-occipital involvement. Nevertheless, it should be stressed that dyslexia exists in every language, even in languages with very shallow orthographies (such as Finnish or Korean Hangul). As a personal example, dyslexics in Finnish, a highly transparent orthography, generally read more slowly and accurately, and those on imaging also exhibit underactivation in left occipito-temporal regions (but the distribution does not appear to be as disproportionately temporo-parietal strain, as phonological decoding is not especially demanding in Finnish). Accordingly, case reports have described a neural hallmark that is less of a traditional phonological bottleneck and more of a failure to automate the visual word recognition process (OTC/VWFA problems).

Demographic fluctuation: In terms of demographic effects, a well-recognized one is the sex prevalence difference (more diagnosed boys than girls, about 3:1 in a school environment), but it may be considered partly a sociological effect (reading problems among boys are selected more easily). As far as the differences in sex in the brain are concerned, the research is actually quite limited and somewhat confused. A few studies in the 2000s indicated that left-hemisphere underactivation in dyslexic boys may be more marked than in girls (who might more commonly process bilaterally), but more recent evidence is ambiguous. Comorbid ADHD in dyslexic boys occurs more frequently and may affect neural results Brimo et al., (2021). The basic neural deficiencies do not seem to vary between sexes; instead, both dyslexic boys and girls exhibit the diagnostic changes in the regular reading network, although the brains of girls appear to have slightly more methods of compensation (a hypothesis that requires further investigation).

The other factor of demographics is age or the degree of development. Dyslexia is a developmental problem, and a dyslexic beginner reader of age 7 will exhibit a different profile of brain activation compared to a dyslexic adolescent of age 15. Dyslexic readers, with maturity and *ad hoc* reading accommodation, typically develop compensatory behaviors. fMRI studies have identified increased anterior reliance (IFG) in adolescent and adult dyslexics compared to children. This often involves a compensatory shift toward right-hemisphere homologs Rollins, (2014).

These developmental patterns are amenable to interventions (e.g., following remediation, there is even more normalization of left-temporal activity). In this way, the picture of neural activity changes over time. The term “cross-sectional studies” used indicates that the influence of development needs to be teased out with reading ages compared to chronological ages.

Bilingualism and culture: There is rising research into the adaptation of the brain of bilingual dyslexics. Combinations of the above patterns may occur in a child who is dyslexic in a language such as English and who also reads Chinese. Bilingual studies in imaging (e.g., Chinese-English speaker dyslexics) have shown that such network deficits may co-exist in the two languages, but a degree of interscript literacy support may also exist. In an extreme example, a bilingual dyslexic could call upon neural pathways used to parse his or her stronger language during the reading of the weaker one, and this might lead to unusual patterns of activation (such as Chinese pictorial strategies circumventing a hang-up on an English word). Such nuances are still at the research frontier, but they speak to the idea that context does matter: The neural defects of dyslexia cannot be said to exist in any vacuum; they are tied to the language environment and the history of learning.

Regarding wider demographic background, socioeconomic status (SES) might affect brain development and may either increase or decrease the effects of dyslexia. Dyslexic children with lower SES may also have aggravating variables (limited opportunity to utilize the intervention, increased stress) that are reflected in neural activity (e.g., overall reduced gray matter or increased stress response ability). Nevertheless, the very essence of the dyslexia neural signature (left hemisphere reading network malfunction) has been confirmed in the successfully diagnosed population of all classes and cultural backgrounds of citizens all around the globe (Table 3).

**Table 3:** Summary of neurostimulation interventions in dyslexia, including target brain regions, stimulation protocols (e.g., tDCS and TMS), outcome measures, and reported short-term reading improvements.

**Study**	**Languages**	**Key neural findings**
Yan et al. (2021) (eLife)	Alphabetic vs. Chinese (morpho-syllabic)	General: The structural and functional deficits within the Left STG in dominating languages under both types of language tasks (universal phonological deficit). Alphabetic: Decreased activation *per se* in the left fusiform (VWFA) and left middle temporal gyrus. Chinese: Decrease of gray mass in the left inferior frontal gyrus (dorsal). Distorted as alphabetic dyslexia, which influences more the orthographic/visual areas, and Chinese dyslexia demands more of frontal/morphological processing.
Li and Bi (2022) (Neurosci Biobehav Rev)	Alphabetic vs. Chinese (children)	These shared and different neural dysfunctions were found. Dyslexics of both systems share underactivation in left temporo-parietal region. There was extra impairment of the visuo-spatial and frontal memory systems in the Chinese dyslexics; extra frontal deficits occurred in the alphabetic dyslexics. Altogether, in line with Yan et al. (2021).
Richlan (2020)	Various alphabetic (deep vs. shallow) and logographic scripts	Putative core left-hemisphere underactivations (VWFA, TPC, IFG) present across dyslexics in all writing systems = universal neural signature. Differences with a dependency on orthography were largely quantitative: e.g., shallower orthographies (Italian, German) produced smaller or more focal clusters of underactivation than deeper orthographies (English) as phoneme mapping was easier. The observed dyslexic underactivity of IFG was not as regular and in some instances showed overactivity according to task and language. Drawing conclusions that the dyslexic brain network is culturally similar in foundation, with the accentuation of the severity/distribution of the demands of the orthography.
Paulesu et al. (2001) (historical)	English vs. Italian vs. French	Initial cross-linguistic PET-scan experiment of dyslexia. English (deep orthography) dyslexics were found to have pervasive hypoactivations in left temporal areas, whereas Italian (shallow orthography) dyslexics had also temporal left hypoactivation although not to the same extent as the other group, since they were mostly afflicted by fluency deficits. They all demonstrated a lower level of activity in the left angular gyrus (TPC). Not in altogether different regions, but in the extent of neural activation: behavioral differences were reflections of accuracy vs. speed impairment. Core deficit + language-modulated severity (Comorbidity with).

Most studies were pilot trials with small sample sizes.

## Diagnosis of dyslexia using technology

**Figure 5:**
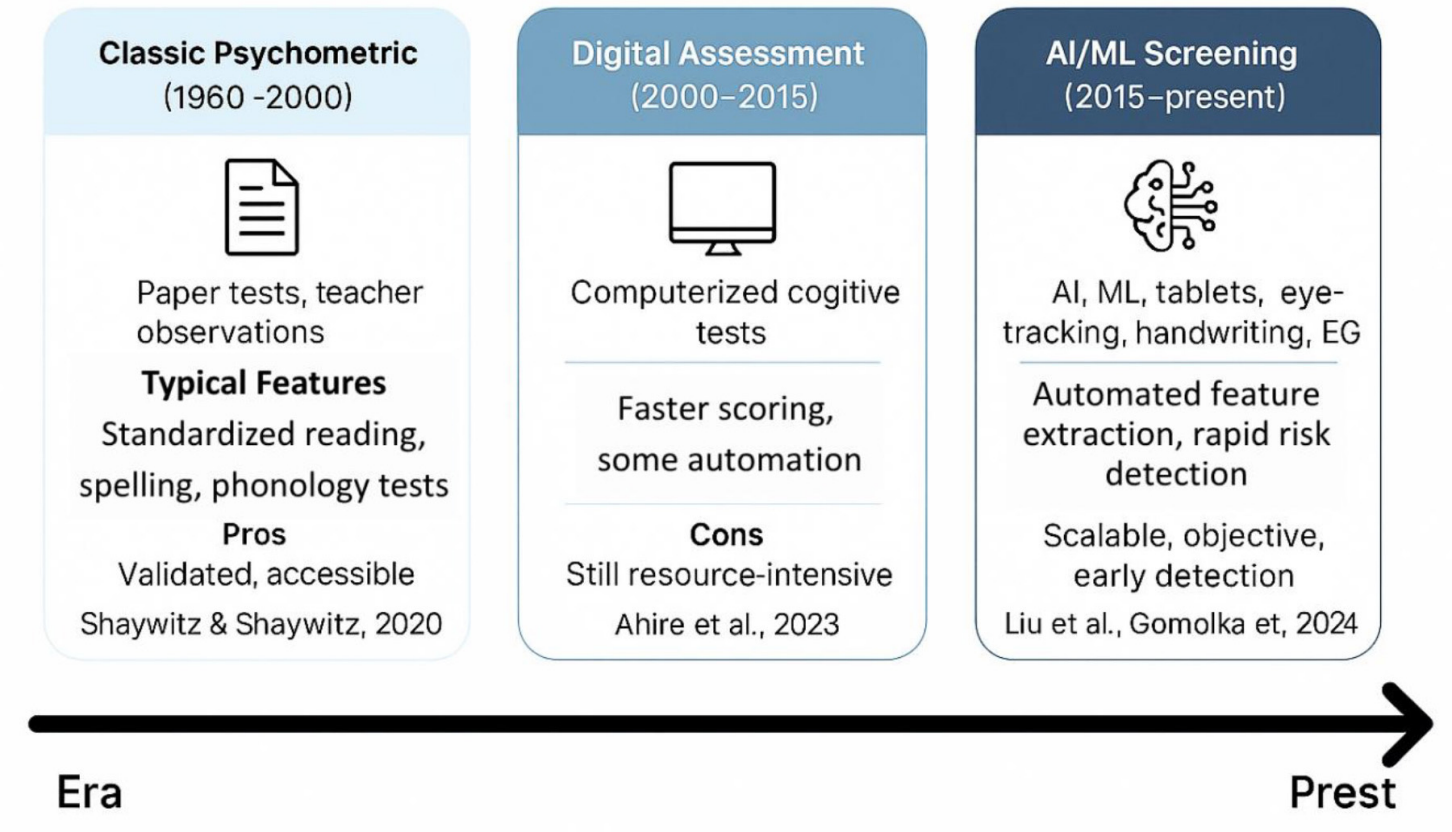
Evolution of dyslexia diagnostics.

The correct and timely diagnosis of developmental dyslexia is one of the key issues in the global community, especially in linguistically diverse and resource-poor environments (Table 4). Conventional diagnostic methods rely on normed reading, spelling, and phonological awareness instruments delivered by trained experts, which are time-consuming, language-specific, and cannot identify even the initial symptoms in at-risk children (Shaywitz and Shaywitz, 2020). Over the past few years, the diagnostics of dyslexia have started to change with the help of technological innovations. AI and ML have become involved to detect these hidden, complicated patterns in behavioral, neurophysiological, and written data, which have previously been difficult to detect by subjective clinical measurement (Ahire et al., 2023; Liu et al., 2024). Such novel breakthroughs provide the prospect of scalable, objective, and more equitable screening and diagnostics, as well as new horizons in more personalized early intervention considering the individual, developmental, and cultural profile (Figure 5).

**Table 4:** Comparison of traditional vs. technology-enhanced dyslexia screening.

**Category**	**Traditional screening**	**Technology-enhanced screening**
Main methods	Reading/spelling tests, interviews	Eye-tracking, handwriting, AI/ML analytics, digital games
Required staff	Trained clinician, teacher	Minimal—can be remote, automated
Language adaptability	Language/test specific	Easily adapted (with data retraining)
Speed	Hours (assessment + scoring)	Minutes (real-time or automated)
Sensitivity	Moderate (late diagnosis)	High (can detect early risk)
Cost	Moderate to high	Low to moderate (after setup)
Scalability	Low (manual)	High (cloud/web or mobile delivery)
Reference	Shaywitz and Shaywitz, (2020)	Liu et al., (2024); Gomolka et al., (2024)

## Machine learning and AI models of dyslexia detection

The methods of machine learning have quickly become popular in dyslexia diagnostics. They involve supervised learning categories utilizing meticulous datasets (for example, procedure information, i.e., read aloud, non-word repetition, quick and controlled naming, composed text, handwriting documents, neuroimaging (EEG, fMRI, eye-tracking), and assessments using electronic games and applications) Ahire et al., (2023); Gallego-Molina et al., (2024). Eye-tracking and handwriting analysis provide reliable indicators for early dyslexia detection. Table 5 outlines the diagnostic performance of recent AI/ML models, demonstrating accuracies exceeding 80% across multiple languages and modalities. These findings align with the conclusions of a recent systematic review, which provides converging evidence for the diagnostic utility of eye-tracking technology in dyslexia detection across different age groups and languages Coenen et al., (2024). All this can be identified given enough data and the right algorithm Liu et al., (2024). Convolutional neural networks (CNNs), recurrent neural networks (RNNs), including long short-term memory (LSTM) networks, support vector machines (SVMs), random forests, and boosting techniques are among the most commonly reported machine learning algorithms that have been used in the diagnosis of dyslexia and, thus, have demonstrated the most promise. A deep learning system, DysDiTect, implementing CNN-LSTM-attention models has been shown to achieve 83.2% diagnostic accuracy using handwriting samples (Liu et al., 2024). These findings are consistent with the broader trends summarized in a recent systematic review, which highlights the growing accuracy and adaptability of deep learning approaches for dyslexia detection and intervention (Kothapalli et al., 2024). These neural networks perform especially well with temporal sequence data, like the dynamics of handwriting or gaze trajectories, in which small temporal irregularities can indicate the presence of neurocognitive deficits (Table 5).

**Table 5:** Performance of recent AI/ML models for dyslexia screening.

**Study (year)**	**Country/ language**	**Model/ approach**	**Data type**	**Accuracy (%)**	**Sensitivity (%)**	**Specificity (%)**	**ROC-AUC**
Liu et al. (2024)	China/Chinese	CNN-LSTM-attention	Handwriting (dictation)	83.2	83.0	83.5	0.90
Gomolka et al. (2024)	Poland/Polish	LSTM network	Eye-tracking	97.7	97.5	98.0	0.99
Gallego-Molina et al. (2024)	Spain/Spanish	Explainable NN (EEG)	EEG during writing task	92.4	93.1	91.7	0.96
Ahire et al. (2023)	Multi/various	SVM, RF, CNN (review)	Multimodal	75–97	70–98	73–99	0.82–0.98
Yan et al. (2021)	Multi/meta	Multimodal ML	Imaging, cognitive, etc.	79–94	74–97	78–96	0.89–0.97

**Figure 6:**
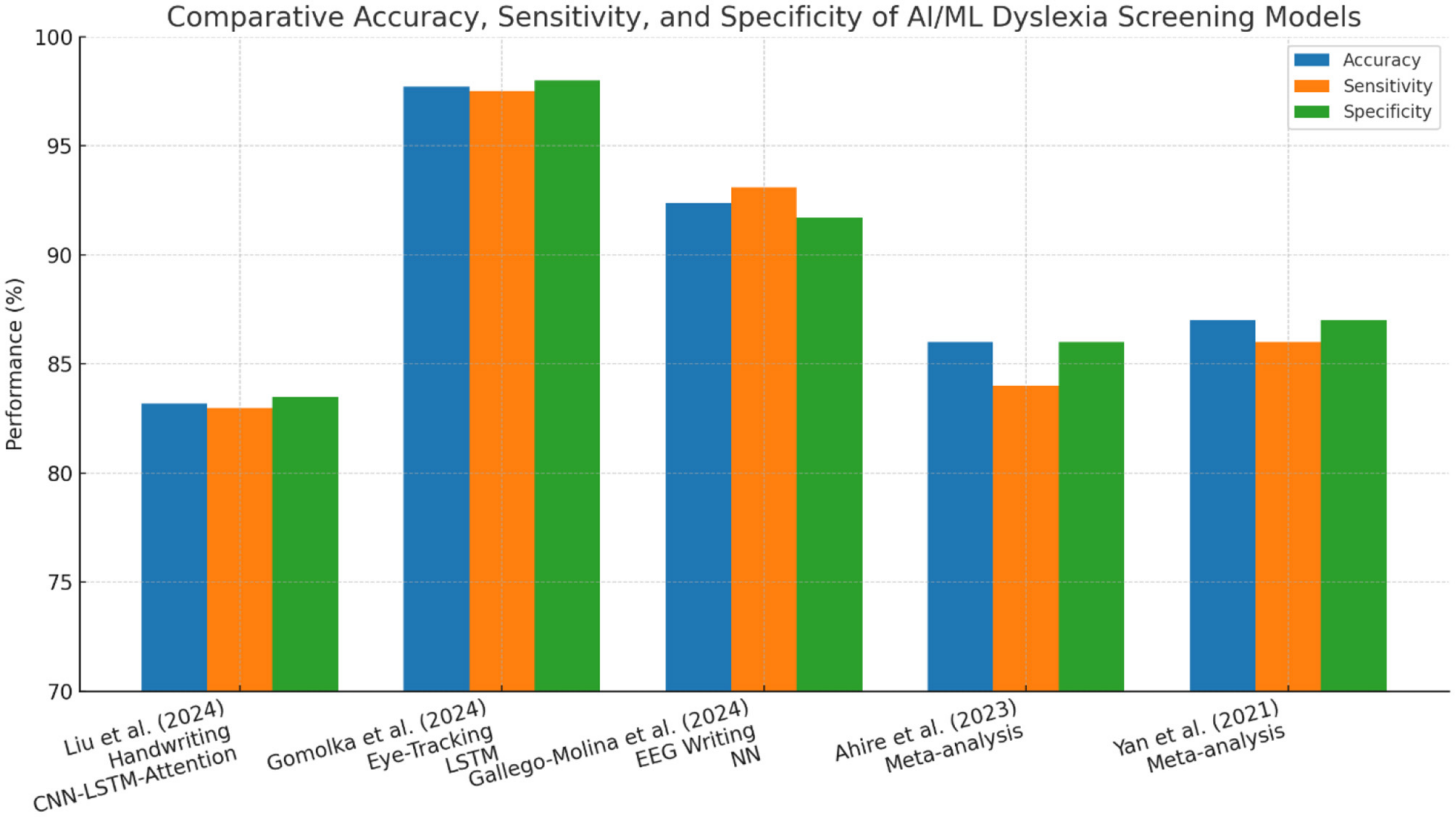
Comparative accuracy for different models in research papers.

Effectiveness of such models is not only reflected in accuracy, but also in high sensitivity and specificity. State-of-the-art systems typically have a performance above 80%, with ROC-AUC values overwhelmingly above 0.90 in recent research (Liu et al., 2024; Gomolka et al., 2024). It is worth noting that an accuracy of 97.7% was achieved using an LSTM model on eye-tracking data from early school-aged children (Gomolka et al., 2024). There is also growing interest in the interpretability of such “black box” models. More recent methods, SHapley Additive exPlanations (SHAP) and gradient-weighted class activation mapping (Grad-CAM), enable the visualization of the features that most powerfully inform model decisions, supporting model trust and interpretability for clinical deployment (Gallego-Molina et al., 2024) (Figure 6). SHapley Additive exPlanations (SHAP) and gradient-weighted class activation mapping (Grad-CAM) are model interpretability techniques. In the context of dyslexia detection, these methods can help identify which input features (e.g., handwriting strokes, gaze patterns) most strongly influence the model's prediction, thereby improving transparency and clinical trustworthiness.

## A handwriting analyst and eye-tracking

**Figure 7:**
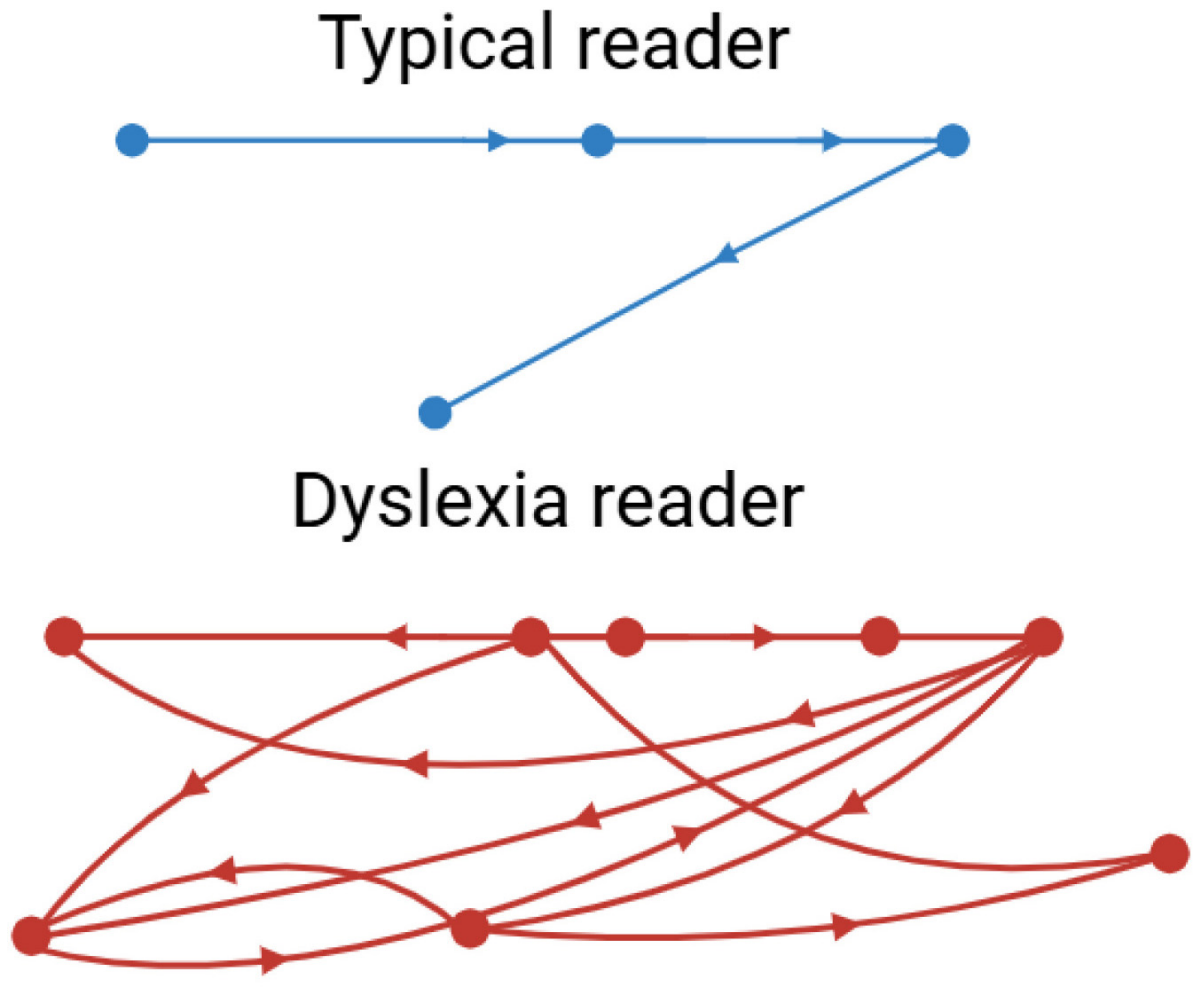
Eye tracking patterns, typical vs. dyslexia readers.

Eye-tracking has become one of the most solid and available behavioral dyslexia diagnostic technologies. Reading is intimately related to the ability to move the eyes efficiently and accurately. Common characteristics of the eye movements of dyslexic readers include extended and frequent eye fixations, smaller saccades, more frequent regressions, and increased overall irregularity and variance in reading eye movements (Toki, 2024) (Figure 7).

Abnormalities in gaze patterns can be detected early and may serve as potential risk indicators for dyslexia, though they are not sufficient for diagnosis on their own. Recent innovations in mobile and remote eye-tracking, the use of ordinary tablets, laptops, or webcams, have highly democratized the use of this type of equipment, allowing mass screening beyond the walls of specialized facilities and research labs. Writing analysis also changes things in similar ways. As people write, particularly using a digital tablet/stylus, higher-resolution data is produced that captures structural and kinematic aspects of letter making, such as stroke pressure, trajectory, and timing. The writings of dyslexic children are usually poorly constructed in terms of the shape of the letters, spacing, variable pressure, and speed, and they include numerous delays at the boundaries between graphemes and phonemes (Dunne, 2015). The analysis of thousands of features in these handwriting samples, possible using modern AI models, can identify patterns that could reveal minor deficits in motor planning, phonological coding, or orthographic representation. A deep learning system has been developed to detect dyslexia using handwriting input (Liu et al., 2024) (Table 6).

**Table 6:** Key eye-tracking features in dyslexic vs. typical readers.

**Feature**	**Dyslexic readers (Mean ±SD)**	**Typical readers (Mean ±SD)**	**Diagnostic value**	**References**
Fixation duration (ms)	300 ± 50	220 ± 30	High	Gomolka et al., 2024
Saccade length (letters)	5.2 ± 1.3	7.8 ± 1.1	High	Gomolka et al., 2024
Regression rate (% fixations)	27.0 ± 6.0	14.5 ± 3.1	High	Toki, 2024
Number of fixations	110 ± 21	78 ± 14	Moderate	Gomolka et al., 2024
Variability (SD of fix duration)	60 ± 15	38 ± 12	Moderate	Dunne, 2015

**Figure 8:**
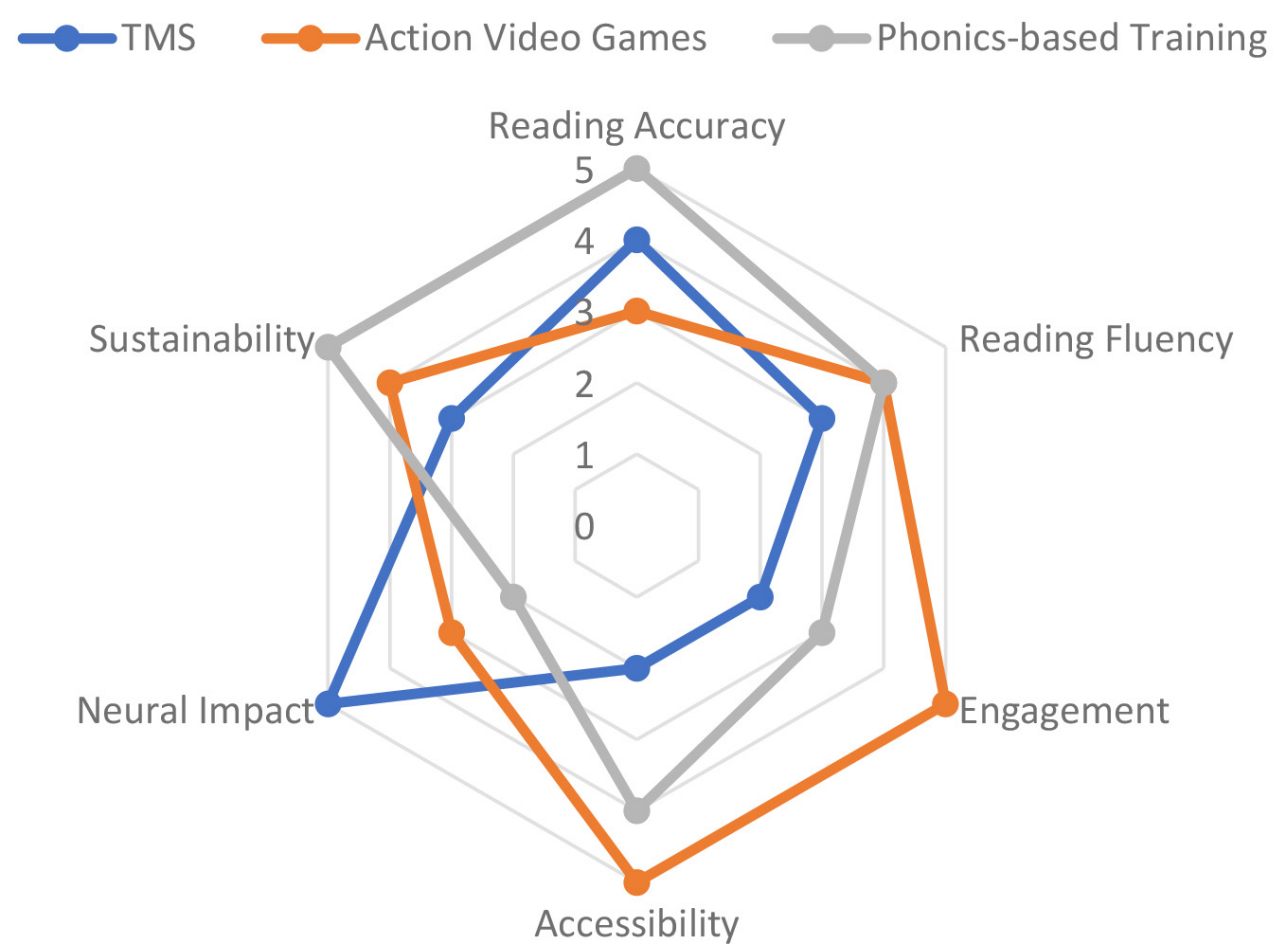
Comparative effectiveness of dyslexia therapies across key criteria.

**Figure 9:**
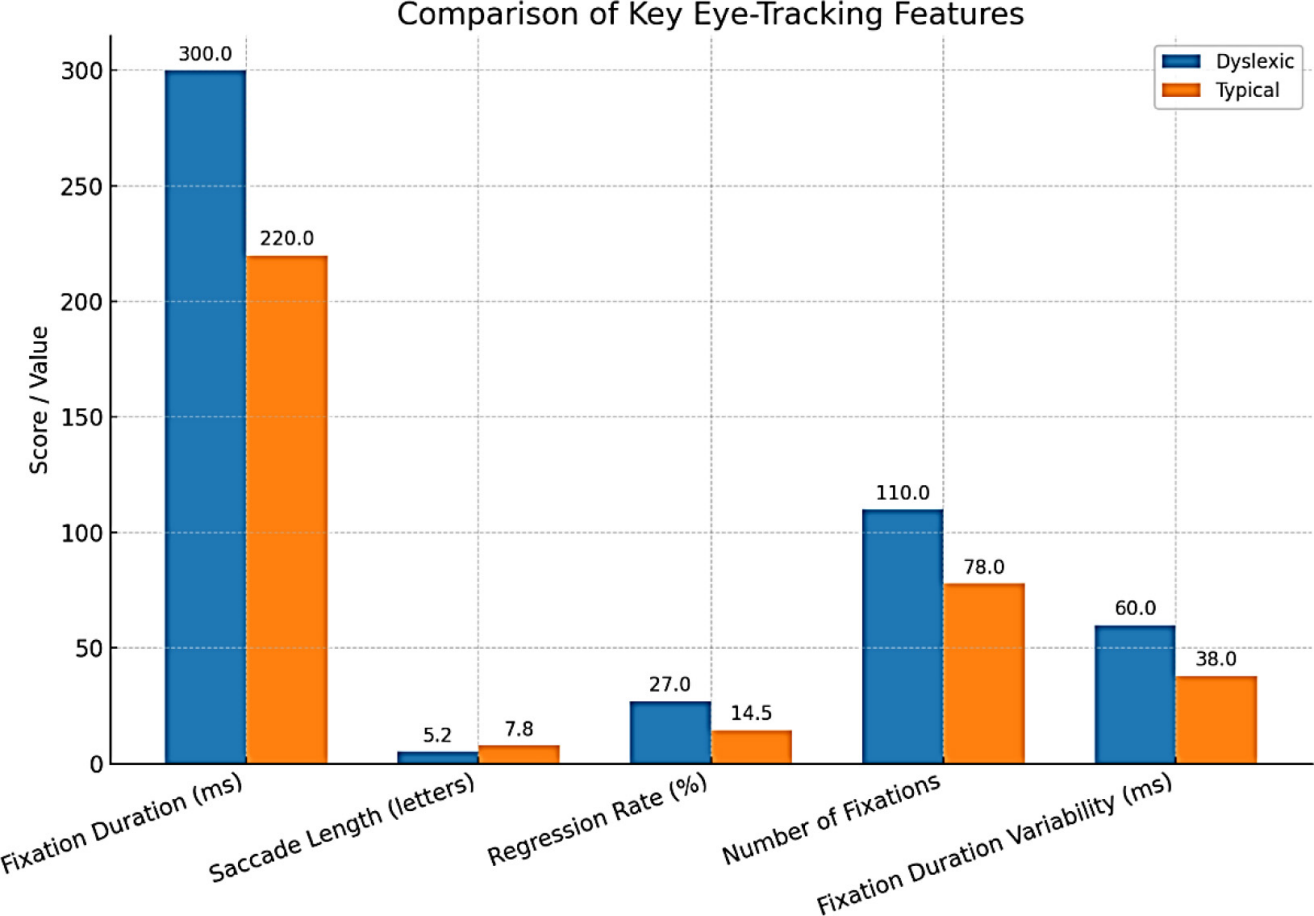
Comparison of key eye-tracking features.

**Figure 10:**
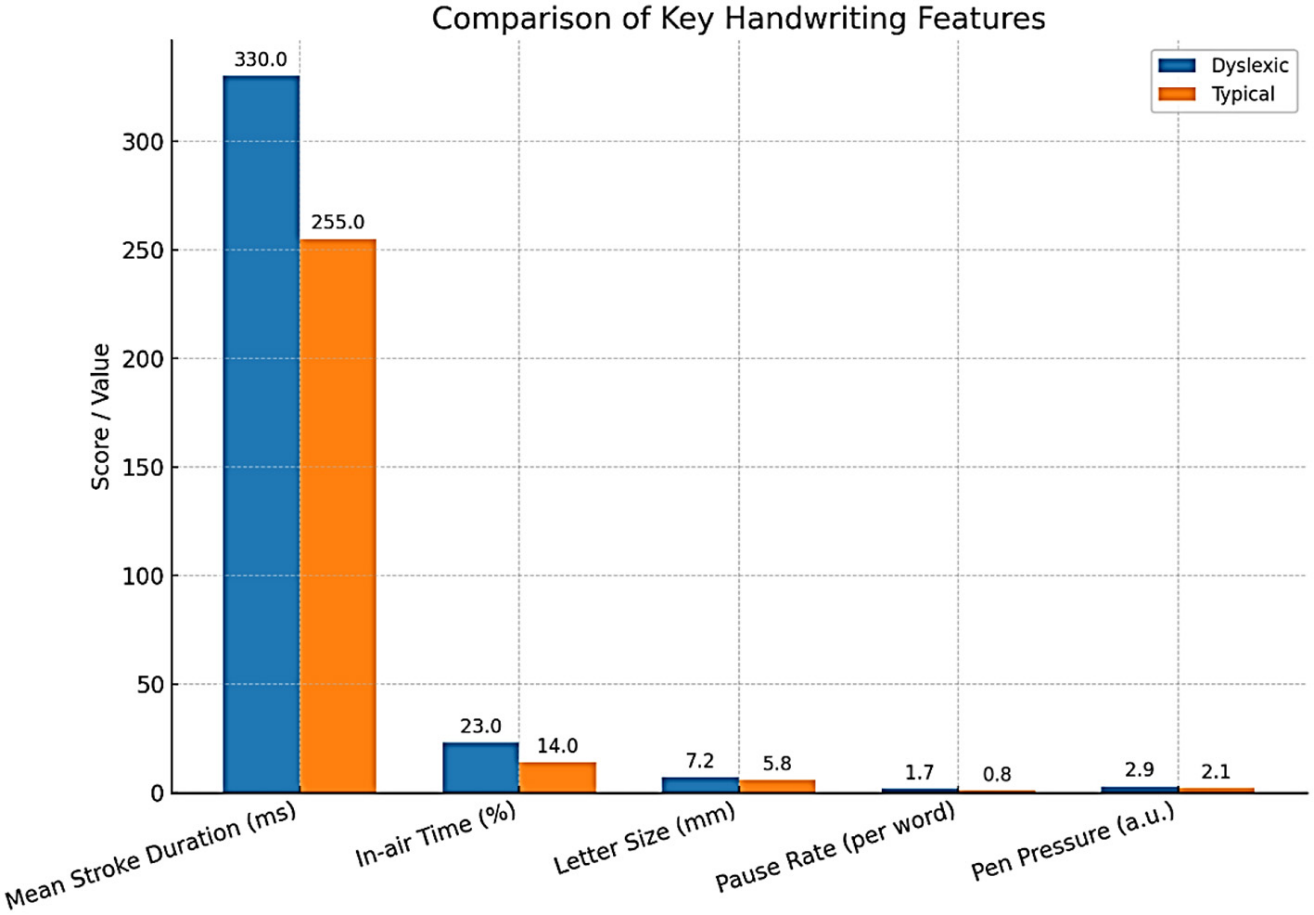
Comparison of key handwriting features.

Eye-tracking and handwriting are gradually being combined with other modalities of computerization (Figures 8–10). The potential of multimodal techniques, i.e., data based on reading, writing, speech, and neurophysiological data (e.g., EEG) at the same time, to better characterize the complexity of dyslexia and offer stronger, more personalized risk models, is particularly compelling (Gallego-Molina et al., 2024).

## Case studies and verification

Validation studies are important for translating AI/ML models developed in academia into effective applications in practice. Some key considerations for the valid and reliable trustworthiness of validation include using large, heterogeneous samples across various schools and regions, prospective designs that can forecast reading problems compared to gold-standard clinical validation, and considering both in-sample and out-of-sample performance (Gomolka et al., 2024; Liu et al., 2024). For example, the DysDiTect model proposed by Liu and others was tested on a group of more than 2,000 Chinese students, with the model demonstrating an error rate of below 20 percent across schools, grades, and assessors. Similarly, the LSTM-based eye-tracking model was tested, and the performance was consistent in both lab and actual school setups, with results showing superior performance even in mixed lighting, background noise, and during group administration.

**Figure 11:**
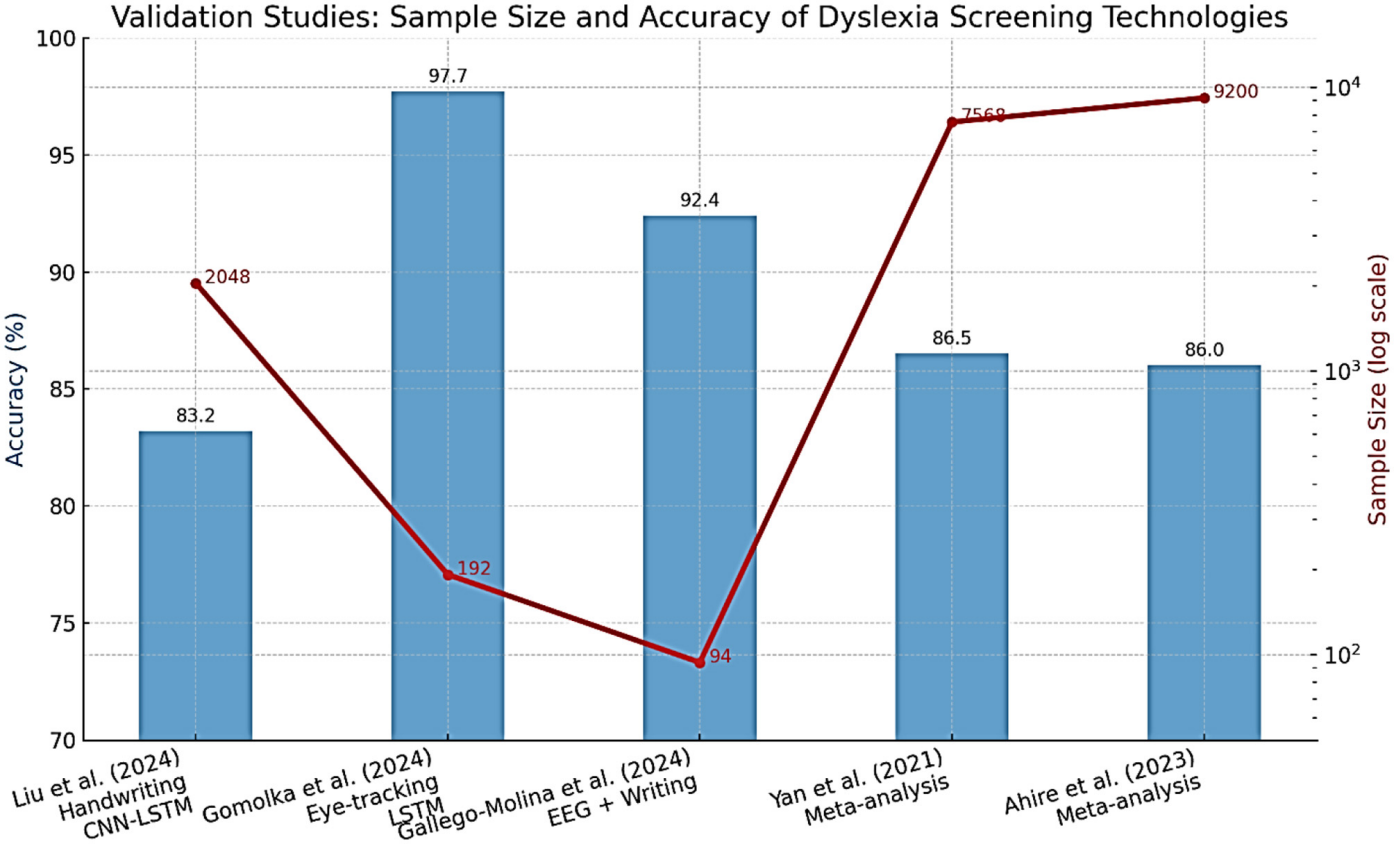
Sample size and accuracy of dyslexia screening technologies.

As shown in Figure 11, sample size significantly correlates with diagnostic accuracy, supporting the scalability of these AI-based screening approaches. Successful large-scale implementations strongly indicate the potential of these technologies for large-scale screening and highlight crucial issues of user acceptance, cost, and ethics. Educators and parents should be confident in the results of diagnostic reports prepared with the help of AI; therefore, visualizing risk factors is important. Another issue is accessibility. Handwriting or eye-tracking-based tablet tests can be low-cost and scalable, making them especially appealing for use in remote or under-resourced schools. However, the sensitive nature of behavioral and neurophysiological data necessitates its safe handling, and developers should routinely test the models to identify potential biases concerning multilingual or non-native populations (Ahire et al., 2023).

## Cross-cultural and linguistic adaptation

**Figure 12:**
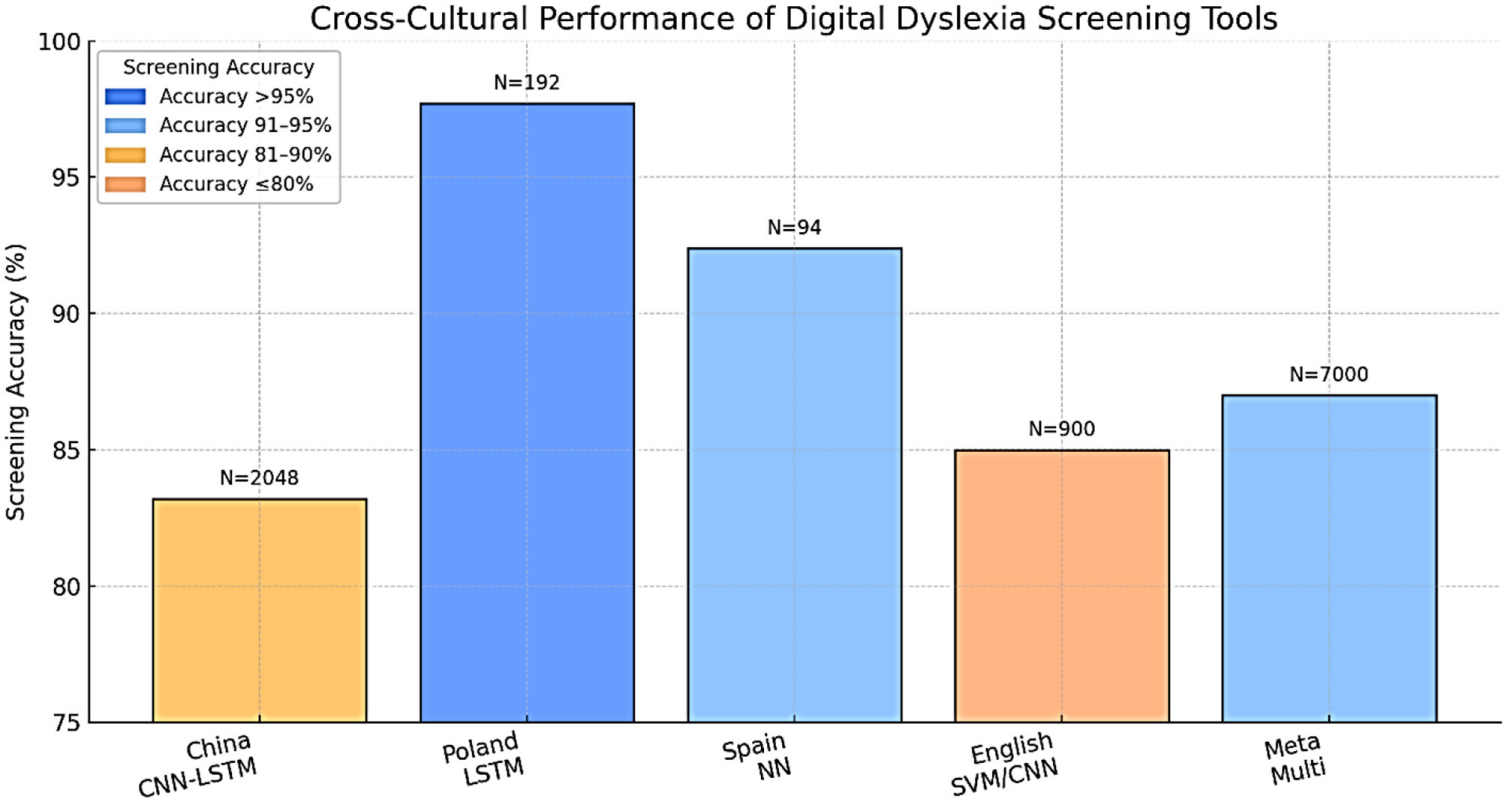
Cross-cultural performance of digital dyslexia screening tools.

One of the strong points of a technologically enhanced diagnostic approach is that it will overcome the old problem of language difficulties. Nevertheless, the form that dyslexia manifests takes into account the writing system (alphabetic, logographic, abugida), orthographic depth, and culture, and requires a careful adaptation of the tool and models to every context (Liu, 2024). Particularly conspicuous are differences in the script. As an illustration (Figure 12), Chinese, being a logographic language, is more demanding of visual-orthographic memory compared to English, which is more dependent on phoneme-grapheme mapping. This implies that AI/ML models are language-specific and cannot just be transferred effectively to another language without being retrained and validated (Johnsen, 2025). Rapid automatized naming is one example of a linguistic feature that can have varying diagnostic utility across languages, and stroke order or spacing features of handwriting can be script-specific. There should also be a discussion of socioeconomic and digital divisions, since not all people have access to necessary gadgets or broadband, especially in lower-income areas (Table 7).

**Table 7:** Cross-linguistic performance of digital dyslexia screening tools.

**Language/ script**	**Tool/ model**	**Main adaptation(s) required**	**Accuracy (%)**	**Sensitivity (%)**	**Specificity (%)**	**Sample size**	**References**
Chinese	CNN-LSTM-attention	Custom character set, stroke features	83.2	83.0	83.5	2,048	Liu et al., 2024
Polish	LSTM eye-tracking	Task/word adaptation	97.7	97.5	98.0	192	Gomolka et al., 2024
Spanish	NN EEG + writing	Task translation, script norms	92.4	93.1	91.7	94	Gallego-Molina et al., 2024
English	SVM, RF, CNN	Language dataset	75–95	74–98	73–99	900+	Ahire et al., 2023
Multi/meta	Multimodal ML	Script-specific retraining	79–94	74–97	78–96	7,000+	Yan et al., 2024

New approaches are being implemented, such as training on several languages, transfer from one related language to another, and tailoring to regional school curricula and student expectations. Close partnership with local teachers and clinicians will be necessary to promote a cultural fit and optimal uptake (Yan et al., 2021). In a single case example, it was proved that properly adjusted AI-based handwriting diagnostics performed very well in relation to both Chinese and English structures. Similarly, testing their eye-tracking system in a variety of Polish schools showed good performance of the system irrespective of whether the schools were located in rural or urban settings (Gomolka et al., 2024).

## Digital and neurostimulation therapies for dyslexia

The intervention design for developmental dyslexia has taken a new turn since the advent of digital therapeutics and neurostimulation of the brain. In the past, interventions focused on direct phonics instruction, multisensory teaching, and individual remedial education. These are still basic, but fast developments in neuroscience, computer science, and mobile technology have introduced a new period of scalable, affordable, and potentially more successful treatment ((Bertoni et al., 2024; Maresca et al., 2022). This section is a synthesis of the state of the art in digital and neurostimulation-based therapies for dyslexia, focusing on the mechanisms of action, protocols, outcomes, and the role of engagement and motivation as being linked to treatment adherence and efficacy.

## tDCS, TMS, and neurofeedback

Several non-invasive brain stimulation methods, especially the use of transcranial direct current stimulation (tDCS) and transcranial magnetic stimulation (TMS), have been examined as possible complements to behavioral reading interventions. The rationale underlying the application of these strategies is the numerous documented brain circuit defects in dyslexia—particularly decreased activation and connectivity of the left temporo-parietal and occipito-temporal areas of the reading system (Richlan, 2020). Using neurostimulation to adjust cortical excitability could facilitate neuroplasticity across these circuits and, therefore, contribute to better reading performance.

## Mechanisms of action

tDCS applies a small, purely electrical stimulus to the scalp, generally by placing the anodal electrode over a target area (e.g., left temporo-parietal cortex) to enhance excitability, and the cathodal electrode over a reference location to attenuate excitability. In comparison, TMS employs quickly moving magnetic fields to stimulate currents in the cortical tissue, which has the ability to stimulate or suppress local neuron groups temporarily. Both methods have been found to evoke changes in local field potentials, oscillatory activity, and functional connectivity that might prime the brain to learn (Rios et al., 2018; Turker et al., 2025).

Another strategy is known as neurofeedback, in which individuals train themselves (typically using EEG to monitor brain activity) to achieve self-regulation by receiving feedback and rewards in real-time on movements toward desired patterns. The protocols of neurofeedback to address dyslexia usually concentrate on improving particular frequency bands or connections between the nodes in the reading network (López-Zamora et al., 2025).

## Clinical trials and protocols

**Figure 13:**
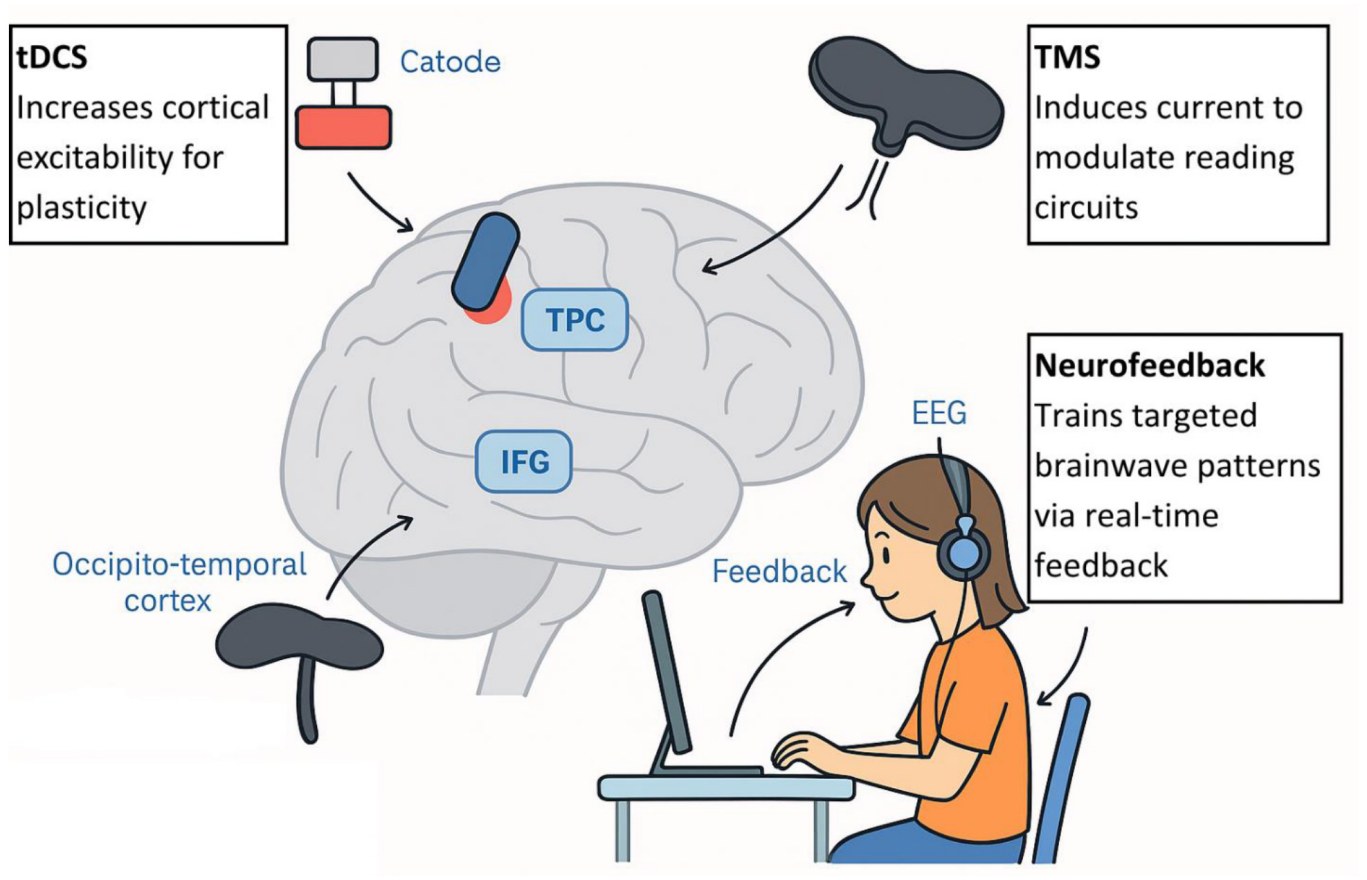
tDCS, TMS, and neurofeedback mechanisms in dyslexia.

A number of neurostimulation protocols have been tested recently in dyslexia research in both children and adults. A randomized controlled trial applied anodal tDCS over the left temporo-parietal cortex to dyslexic children in combination with reading training (Turker et al., 2025). These studies demonstrate promising efficacy and safety of neurostimulation interventions in children. A systematic review also supports the use of TES interventions in children and adolescents, demonstrating improvements in phonological processing and reading fluency (Fathi Azar et al., 2024). In effect, the treatment group realized substantial improvements in measured reading fluency and phonological awareness outcomes over sham stimulation, and outcomes remained sustained at three-month follow-ups. Similarly, the use of tDCS on the left inferior frontal gyrus has been shown to produce significant improvements in reading speed and accuracy (Rios et al., 2018). Although fewer data exist, TMS studies are also positively promising. For example, there is evidence that short trains of repetitive TMS over the left posterior temporal cortex are linked to temporary gains in pseudoword decoding and phonological processing in both adults and children with dyslexia. Neurofeedback studies are still new, and limited studies show that training coherence in the beta band (related to attention and cognitive control) can encourage reading gains, but the results are inconclusive, and clinical trials are required (Figure 13).

## Safety and results

Studies indicate that neurostimulation is capable of eliciting moderate, statistically significant effects in reading accuracy, fluency, and phonological processing among dyslexic patients, particularly in combination with behavioral therapy (Maresca et al., 2022; Turker et al., 2025). Effect sizes of tDCS-enhanced reading training are reported to be within 0.5 and 0.7 SD of the effect size for reading speed and accuracy. Nevertheless, not every study reports strong effects, with the variation in response between individuals being quite high. When tDCS and TMS are used with protocols, safety profiles are attributed to be favorable, even in pediatric populations. The majority of side effects are minor and temporary (e.g., tingling of the scalp, a headache). Nevertheless, ethical concerns and the necessity of professional supervision are worth considering, at least in the case of interventions in young children or beyond the confines of controlled research (Salles and Farisco, 2024) (Table 8). These results are in line with recent systematic reviews showing that transcranial electrical stimulation can enhance cognitive and academic skills in adults with specific learning disabilities (Azar et al., 2025).

**Table 8:** Representative neurostimulation trials in dyslexia.

**Study (year)**	**Modality**	**Target region**	**Age group**	**Protocol**	**Main outcomes**
Turker et al. (2025)	tDCS	Left temporo-parietal	8–12	20 min x 5 sessions	Reading fluency, phon. awareness
Rios et al. (2018)	tDCS	Left IFG	10–14	15 min x 10 sessions	Reading speed, accuracy
Hernández-Vásquez et al. (2023)	TMS	Left post. temporal	9–15	rTMS, 10 min, 5 Hz	Pseudoword decoding (short-term)
López-Zamora et al. (2025)	Neurofeedback	Parietal/temporal	7–13	30 min x 10 sessions	Reading (mixed results)

## Digital interventions

**Figure 14:**
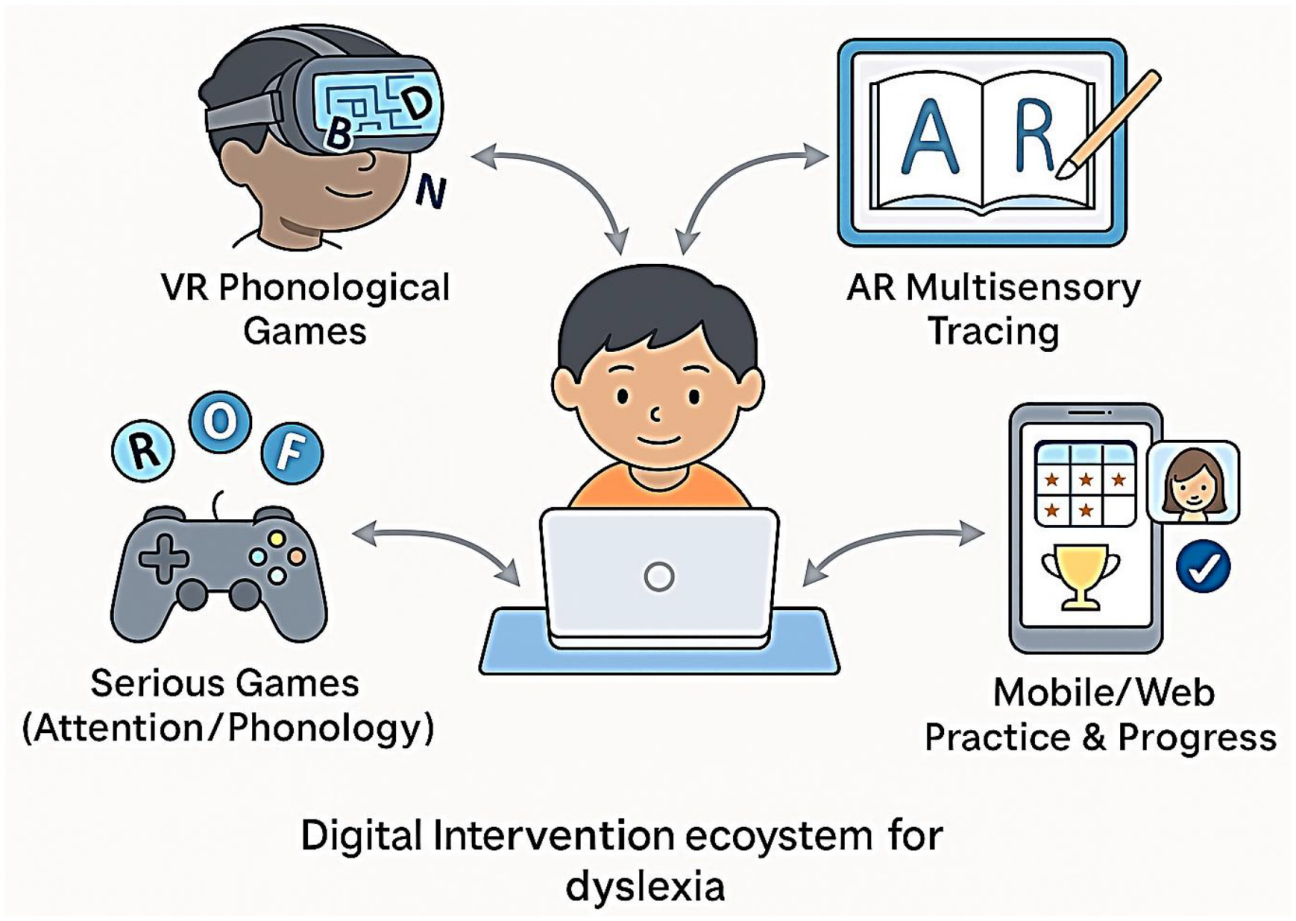
Digital intervention ecosystem for dyslexia.

The treatment of dyslexia is fast being transformed by digital therapeutics, which include virtual reality (VR), augmented reality (AR), and game-like experiences (also called serious games), as well as mobile and web-based solutions that provide intensive, adaptive, and engaging practice to more children (Bertoni et al., 2024; Maresca et al., 2022). This is further supported by a systematic review showing that serious games produce significant improvements in literacy outcomes for children with learning disabilities, particularly when adaptive feedback is integrated (Marinelli et al., 2023). These methods employ the effectiveness of immersive settings and instantaneous feedback to strengthen inspiration, engagement, and educational success (Figure 14).

## Augmented and virtual reality (AR/VR)

VR-based interventions allow users to be exposed to simulated settings where reading activities can be gamified. As depicted in Figure 14, these interventions form part of a broader digital therapeutic ecosystem that enhances engagement and reading outcomes in dyslexic children. To illustrate, VR programs can include going through a word maze, matching letter–sound combinations, or constructing words to break down a virtual barrier. VR therapy has been shown to produce significant positive effects on phonological awareness and word decoding in dyslexic children, with improvements persisting at the 2-month follow-up (Maresca et al., 2022).

AR tools combine interactive reading lessons with reality through the use of tablets or smart glasses. This can be used effectively in reviewing the correspondence of letters to sounds or multisensory letter tracing. AR has individualized, context-aware reading potential at an early stage of development.

## Action games

Action (or, sometimes, therapeutic) games are specifically created video games that teach cognitively, linguistically, or attentively related skills for reading. These are not, however, the play-based commercial entertainment games; more likely, they include adaptive difficulty, corrective feedback, and celebration of progress. One benchmark experiment by Bertoni et al. (2024) demonstrated that training with action video games normalized phonemic awareness and enhanced the reading performance of pre-readers with a family history of dyslexia. In the study, the children who played specially designed games showed not only normalization of neural markers (measured by EEG) but also behavioral benefits. These benefits did not just manifest in performances in the game but also in aspects of standardized reading tests—a very important practice in real-life applications. In other action games, the game is aimed at visual attention span (e.g., identifying letters rapidly), rapid automatized naming, or working memory. According to research, game-based interventions, especially for children who fail to respond to traditional phonics training, are very efficient (Maresca et al., 2022).

## Interventions on mobile and web-based

Dyslexia remedial apps on smartphones and iPad tablets are now universal. Most of them include evidence-based instructional patterns (phonics, decoding, comprehension) containing gamified feedback and adaptive advancement. Lorusso et al. (2024) tested a web-based online treatment program, with the results showing that online delivery was capable of matching, and even in some cases outperforming, face-to-face delivery when considering reading and spelling improvement, becoming particularly effective when children are highly involved.

The mobile applications make it possible to practice at home, involve parents, and allow frequent (high frequency, low dose) sessions, all of which have been shown to foster neural plasticity and the learning of a skill (Table 9).

**Table 9:** Examples of digital and game-based interventions in dyslexia.

**Intervention type**	**Platform**	**Key feature(s)**	**Sample finding/ outcome**
VR word maze	PC/VR headset	Immersive, adaptive phonics	Phonological awareness (Maresca et al., 2022)
Action video game	Tablet/ console	Rapid attention, phoneme link	Phonemic awareness, neural normalization (Bertoni et al., 2024)
AR letter tracing	Tablet/AR	Real-world multisensory	Letter-sound mapping (pilot data)
Web remote program	Computer/ tablet	Daily practice, parent report	Maintains/increases reading (Lorusso et al., 2024)

## Plasticity mechanisms

**Figure 15:**
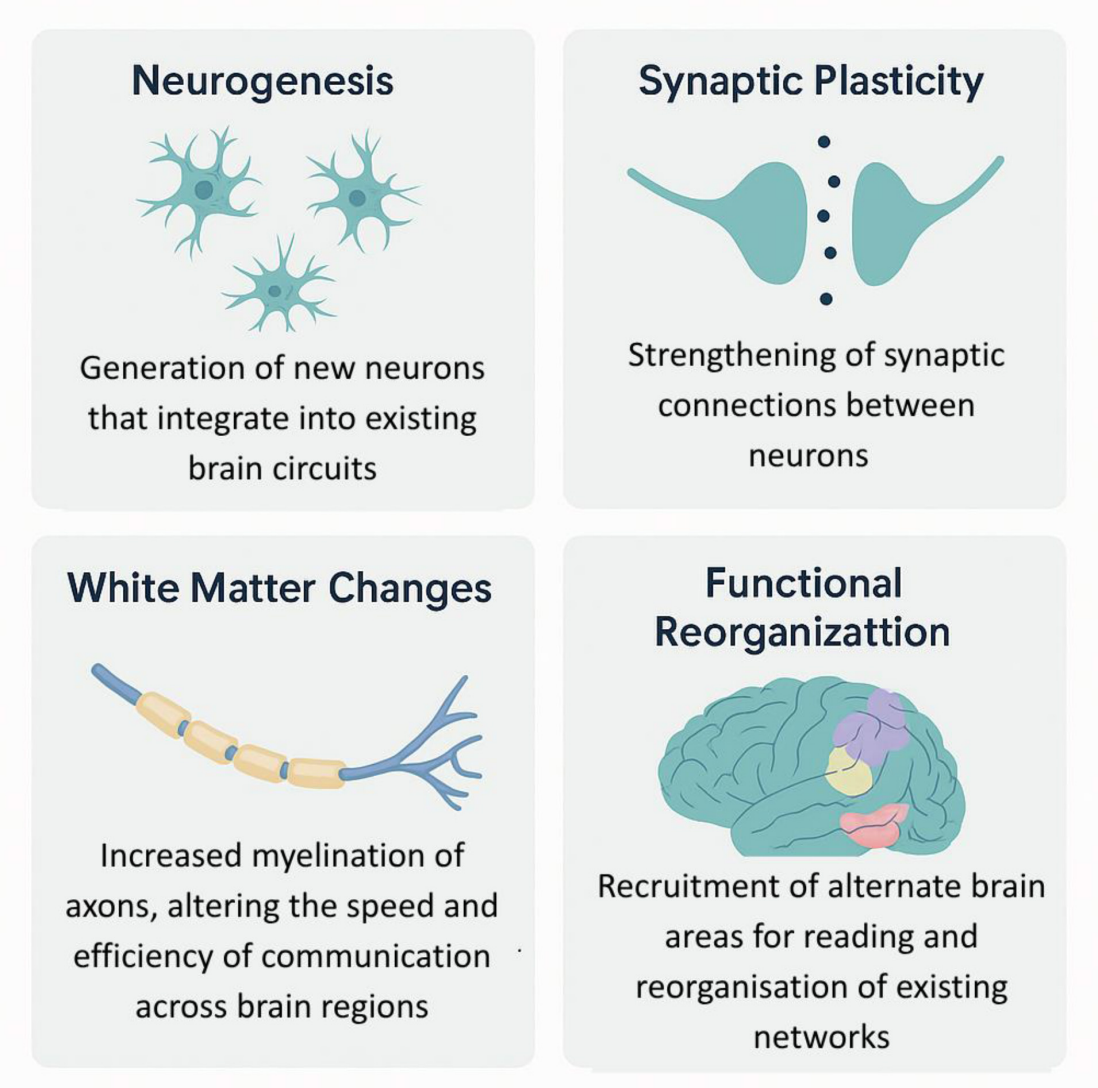
Mechanisms of brain plasticity in response to reading interventions.

One of the shared assumptions of neurostimulation and digital therapeutics is that the brain at work with reading is plastic: it is able to rearrange and reinforce its neural networks through purposeful repetition (Zhang et al., 2023). The basis of such increases in children with dyslexia after intense intervention has been shown to be located at the level of plasticity and is seen in reductions in behavioral, structural, and functional changes at the neural level (Kershner, 2021). As demonstrated by using functional MRI and EEG, the probabilities of successful intervention work have to do with the enlargement of the left hemisphere reading areas' activation, normalization of neural oscillations, and strengthening the interconnections of the occipito-temporal, temporo-parietal, and inferior frontal gyrus (IFG) nodes. Consider, as an example, experimental work showing that an action video game training program in pre-readers not only enhanced behavioral phonemic awareness but also shifted EEG activity toward normal developmental patterns (Delavari et al., 2025). It increases the integrity (fractional anisotropy) of major reading tracts with white matter imaging (DTI) following effective intervention (Meisler et al., 2024). Such neural alterations are not maintained at the end of the intervention, which implies that digital and neurostimulation interventions are ways to provoke long-term circuit rewiring for reading (Figure 15).

Motivation and engagement of a child plays a critically important role in the efficacy of any intervention. Digital and gamified therapies have an advantage over others, however, because they provide instant feedback, recognition, and escapist rewards with different challenges to keep them engaged (Sinha, 2021).

The most important mechanisms are:

- Adaptive difficulty: Maintaining activities in the zone of proximal development for the child.
- Instant response: Motivation to work hard and help lower frustration.
- Goal setting and rewards: Incentives for persistence and a positive effect.

**Figure 16:**
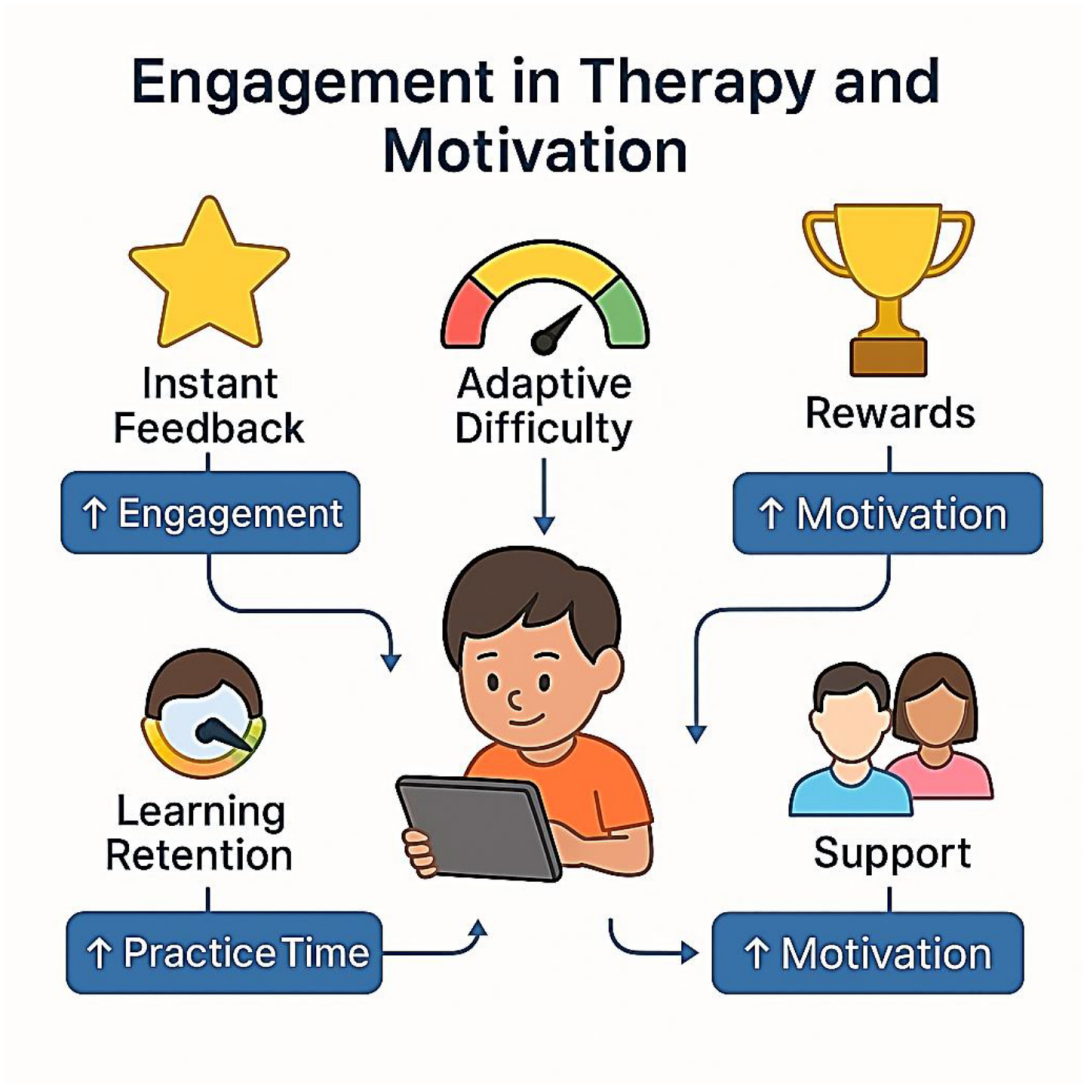
Engagement in therapy and motivation.

Nevertheless, we must be sure that the use of the digital format can be translated to effective learning and transfer to actual reading. Extrinsic rewards used too much or in games not designed well can curtail intrinsic motivation, and therefore, instructions occurring in design are very important. Generalization can also be engaged and reinforced further through parent and teacher input, achieved with the help of monitoring and distance communication (Figure 16).

## Comparative efficacy: traditional vs. tech based interventions

The search for the best way to intervene in dyslexia has taken different leaps in the last 20 years. Although the old methods of treatment for dyslexia, such as explicit instruction based on phonics, multisensory structured language teaching, and individual remedial tutoring, still constitute the core of dyslexia therapy, the development of technology-based interventions has opened new horizons in the areas of scalability, customization, and engagement (Wang et al., 2021). More and more, researchers, clinicians, and policymakers are posing questions about such digital and neurotechnological methods in comparison with best-practice conventional methods. What are the best populations served by each, and are there any benefits to hybrid models? In this section, the authors synthesize the current evidence to compare the efficacy, durability, and inclusivity of technology-based interventions for dyslexia with those based on traditional methods.

## Meta-analysis

Meta-analytical research offers a stringent perspective used in contrasting the effectiveness of interventions across a range of techniques and communities. High-quality meta-analyses testing both conventional and technology-based interventions addressing dyslexia have exploded in the past 5 years. Conventional methods, which are based on Orton-Gillingham, phonics, and multisensory instruction, demonstrate moderate to strong effects in helping develop decoding, word recognition, and fluency in early elementary school-aged children (Galuschka et al., 2014). Indicatively, an average effect size (Hedges g) of 0.65 was reported in reading interventions among school-aged dyslexic children based on the review conducted, with maximum gains achieved in interventions focused on teaching systematic phonics and high-frequency practice.

More recent meta-analyses of digital and neurostimulation interventions provide more encouraging (but somewhat more variable) findings. In a meta-analysis of 21 digital treatments, including VR, serious games, and app-based interventions, a pooled effect size of 0.54 for reading accuracy and 0.58 for fluency was reported, with significantly larger effects observed in interventions incorporating adaptive feedback and gamification (Maresca et al., 2022). Importantly, some digital interventions that encompassed robust aspects of learner-paced, real-time feedback, and multimodal input (e.g., using a combination of text, audio, and interactive assignments) showed great outperformance when compared to static, non-adaptive platforms (Table 10).

**Table 10:** Key findings from the meta-analysis.

**Author (year)**	**Intervention type**	**No. of studies**	**Avg. effect size (g or d)**	**Key findings**
Galuschka et al. (2014)	Phonics/multisensory	36	0.65	Systematic phonics are most effective
Maresca et al. (2022)	Digital (apps, VR, games)	21	0.54 (accuracy), 0.58 (fluency)	Gamified/adaptive tools are best
Turker et al. (2025)	tDCS/TMS (with training)	11	0.4–0.7	Best with paired behavioral therapy
Lorusso et al. (2024)	Traditional vs. digital	1 (RCT)	NSD	Comparable long-term gains

NSD, No significant difference.

## Sustainability and long-term results

The maintenance of reading over time after the intervention is an essential objective of any given therapy. Most traditional interventions tend to describe strong effects in the short term, with a tendency for fade-out in the following year in the case of non-practice maintenance. Clients with preserved skills often require booster sessions and reinforcement at the family/school level.

On the other hand, digital interventions have promising findings regarding durability, especially when they include long-term engagement used to immediately assess participants, monitor their progress, and engage parents (Javed et al., 2024). Distributed practice and flexible scheduling, as well as post-intervention access, are possible via web and application-based platforms. Cattoni (2022) also found that children who continued using VR and gamified reading apps at home continued to improve their reading abilities 2 and 6 months after the intervention commenced, further enhancing their skills in comparison to their peers who did not continue to use the intervention.

Another limited but growing consensus is that hybrid models (a combination of the key components of evidence-based instruction—explicit phonics and scaffolded comprehension—with digital implementation, neurostimulation, or gamified practice) may offer the most sustainable effect, at least for children at risk of dropping off when working with traditional therapies (Par, 2021).

## Subgroup analysis: comorbidity, SES, age, and others

The most critical question that could be asked in comparative efficacy studies is the question, What works for whom? Dyslexia is a non-homogeneous condition, with great dispersion in cognitive phenotype, co-morbidities (e.g., ADHD and DCD), socio-economic status and language, and age of treatment (Table 11).

**Table 11:** Traditional vs. tech-based interventions.

**Moderator**	**Effect on traditional interventions**	**Effect on tech-based interventions**	**References**
ADHD/comorbidity	Moderate gains; may need extra support	Greater gains with neurofeedback, games	Bertoni et al., 2024
Low SES	Limited access/retention	Improved reach with remote/mobile	Lorusso et al., 2024
Early age	Larger effect sizes	Larger effect sizes	Tilanus et al., 2016
Adolescents/adults	Gains possible but smaller	Gains possible with tailored digital	Turker et al., 2025
Language/script	Effective with adaptation	Effective with adaptation	Yan et al., 2021

## Policy, equity, and accessibility

Regardless of the increased potential of AI-driven diagnostics, virtual interventions, and neurotechnological interventions for dyslexia, access is not equally available along geographic, socioeconomic, and cultural lines. Though these tools are extremely effective at both research and clinical levels, they are not always implemented in real-life educational systems due to infrastructural, economic, and linguistic constraints. This gap presents critical consequences for the fair diagnosis and treatment of dyslexia, especially in areas that are already disadvantaged by conventional educational and health systems. The incongruity between high prevalence and poor access is especially evident in low- and middle-income countries (LMICs) (Singh, 2025). Cases of dyslexia may occur in as high as 30 percent among children in some regions, like Nigeria and parts of the Middle East, but the educational systems in these places hardly have any kind of early diagnosis, to say nothing of AI-assisted diagnostic platforms (Ngwu and Nuhu, 2022). The obstacles are varied: poor internet penetration, lack of access to devices, insufficiently trained teachers, and no content adapted to non-dominant languages or writing systems. Low bandwidth, as well as digital illiteracy among caregivers and educators, hinders the adoption of either mobile- or web-based tools, even in areas where access to smartphones is available. Inequality does not only occur in LMICs; in the high-income economies of the United States and the UK, child screening using AI-assisted tools is much less likely to be accessible to low-income, minority, or rural communities than it is to more urban or affluent populations, according to recent audits. This digital inequality is further worsened by structural problems: underfunded schools, a lower number of trained support personnel, and policies that vary significantly among districts, as opposed to consistent national directives. These effects can be visualized by cross-national comparisons of coordinated policy. Finland has effectively integrated the use of digital tools for screening and intervention into its national education system and finances it centrally, as well as trains its teachers. Chile has introduced countrywide tablet-based screening in poorly served schools, and the materials are translated into native tongues (Zugarramurdi et al., 2022). Contrarily, while the national education policy recognizes learning disabilities, it lacks the means to extend digital tools to the rural schools where most of the population is concentrated, leaving it unable to correct the situation. These comparisons indicate that law (or policy), as opposed to national income alone, defines the extent of integration and availability of tech-assisted dyslexia care. The laws tend to be out of pace with innovation. Although more than 40 nations have a legally recognized cause for dyslexia through the dyslexia learning disorder, they have not incorporated the aspect of digital learning and support into their national systems. Legacy models employing in-person evaluations and a one-size-fits-all approach are clumsy, slow, and prohibitively expensive, and many of these systems continue to rely on them. In fact, in cases where they pilot AI and digital technology, such as in the U.S. or UK, it is not always available due to ambiguous regulatory frameworks or budgetary constraints. Consequently, some of the most beneficial technologies can be accessed by the children who require such benefits the most (Mather et al., 2020).

There are additional difficulties with language and cultural adaptation. The majority of dyslexia detection and therapy AI models are trained on well-resourced languages like English, Mandarin, or Spanish. Adapting these tools directly to other linguistic situations, without retraining, runs the risk of significant loss of precision. An example of this is that models used to write and process speech in an alphabetic script may actually fail to transfer to a logographic script or abugida script (Shalileh et al., 2023). There can also be variations in the relevance of certain diagnostic tasks between languages; an example would be tasks of rapid naming or manipulating phonemes. There is a danger of underdiagnosis and misdiagnosis in diverse linguistic populations without specially targeted adaptation. There is innovation with inclusive models in some countries. In Kenya, a tablet-based screening tool in Swahili has been implemented in lower-income schools, incorporating visual and phonological tasks relevant to the local context (Heinrich et al., 2020). South Korea has fully embraced VR-based therapeutic devices in their state-owned schools, and this is supported by the entire government guaranteeing equal opportunities in both urban and rural parts of the country. These projects emphasize the possibility of scalable and equitable implementation of digital solutions in the process of co-creation with policymakers and educators. The data provided on a global level show that coverage continues to vary in dyslexia screening. Although Scandinavian countries screen more than 90 percent of children before they turn 7, in much of Africa and Southeast Asia, virtually none are screened (Torsheim et al., 2010). Domestically, among the lowest income quartiles of children, where differences are greatest, the likelihood of getting an early diagnosis is reduced by as much as five times that of wealthier children. Such disparities exist not only on the infrastructural level but also intertwine the priorities of educational planning and general health policy of entire communities. Unless equity indicators, like the income bin that measures access rates or linguistic inclusivity, are tracked systematically, there is little accountability in ensuring that tech-based solutions, in fact, work to overcome the diagnostic divide.

Although digital technologies promise to individualize and scale dyslexia care, they only succeed in the ecosystems where they exist. The so-called cartridge interventions are not particularly effective when used in isolation, especially in environments where there is little support in the form of parental involvement, teacher training, or alignment of the curriculum. Research has demonstrated that different results may be achieved when the same tablet-based intervention is applied to a school with trained teachers compared to one with caregivers provided with orientation on the use of the tool. Therefore, accessibility should be understood not only as the given technology being available but also as the intersectionality of infrastructure, policy, training, and relevance to the cultures. Equity in technology-mediated dyslexia care will also be achieved through a layered strategy: the enactment of specific legislation to require early screening and to accept the validity of digital tools as an intervention channel; investment in quality infrastructure (connection and device) availability; and funding models that prioritize high-risk or underserved groups. Additionally, to provide culturally targeted diagnostic and therapeutic platforms, one must actively cooperate with linguists, educators, and the local population. In the absence of these measures, there is a danger that technological innovation will be used only to benefit the most privileged, widening existing educational disparities instead of remedying them.

## Future directions in dyslexia

New approaches to dyslexia treatment take the form of hybrid models using neuroscience-based treatments and educational tools with AI capabilities. An example of similar studies is the change in the connectivity of brain networks through neurofeedback (EEG-based brain training) combined with targeted visual exercises in children with dyslexia (Taskov and Dushanova, 2022), which provided neurobehavioral evidence of the exquisite brain functional plasticity that researchers can achieve through integrated neurotechnology and visual training. Similarly, a 30-session EEG neurofeedback intervention was shown to increase reading accuracy and comprehension and to normalize dyslexia-related dysfunctions in coherence patterns, effects that were not observed in the sham practice (Albarrán-Cárdenas et al., 2023). These results imply that real-time feedback on brain signals could enhance the benefits of cognitive training. Immersive technologies are also in pilot mode: a recently developed gamified VR/AR-enhanced reading intervention for primary dyslexic students has shown promising trends in language and executive function, as well as in motivation and satisfaction levels.

Meanwhile, there is a blending of adaptive AI tutors and assistive applications with normal instruction. Paglialunga and Melogno (2025) discovered that personalized adaptive learning systems and game-based AI tutors were the two most popular interventions used in recent investigations, and all of them showed excellent results in reading and arithmetic performance. For example, one of the studies claimed a guided reading ChatGPT tutor yielded very large increases in comprehension compared to regular training. Text-to-speech readers and other assistive applications have also demonstrated high efficacy; for instance, one speech-synthesis application (Speechify) increased the reading retention scores of dyslexic students by more than 15 points compared to other alternatives (Akase et al., 2023). Such composite interventions involving AI-powered customization, gamification, neurofeedback, and multimedia instruction describe a translational neuro-educational paradigm: integrating lab-based findings and the classroom. Importantly, the creation and delivery of such models need to be done with the robust cooperation of neuroscientists, cognitive psychologists, teachers, and technologists. Interdisciplinary teams that can align brain-based practices with pedagogical theory and curriculum requirements are necessary. Consistent with this, Alkhurayyif and Sait (2024) stress that “interdisciplinary collaboration is a prerequisite” to develop multifaceted solutions to dyslexia by integrating neural, behavioral, linguistic, and educational points of view.

Although technically optimistic, the use of technology in dyslexia treatments has an immature evidence base. The vast majority of published research is small, short-term, and methodologically weak. None of the ongoing intervention trials on AI are considered to be of low risk of bias, and although the effects are large, conclusions should be drawn with caution, as Paglialunga and Melogno (2025) also observe. The essential gaps comprise the absence of randomized controlled trials with sufficient sample sizes, and above all, the near absence of long-term follow-up. High-quality RCTs and long-term longitudinal studies following students months or years after the end of an intervention (thereby showing true learning—skill acquisition and retention—rather than a short-lived “performance illusion”) should be the future priorities of research. Long-term evaluation is particularly vital since there are concerns (noted in general learning tech) that shortcuts in the form of easy AI support can artificially boost short-term scores and fail to develop high levels of competence.

The open science practices should also be embraced by researchers in order to expedite the process. Alkhurayyif and Sait (2024) believe that it is possible to improve transparency and reproducibility in the research of technology to treat dyslexia by sharing data and code and using tools to collaborate. Standardized and publicly available sets of data on dyslexia (e.g., based on annotated EEG or eye-tracking) would permit the fair comparison of AI algorithms and stimulate innovation. The development of community challenges or workshops (as would be done with medical imaging competitions) might create cross-team work. Furthermore, core outcome measures of dyslexia interventions should be established by interdisciplinary consortia to ensure that the studies are comparable. Nowadays, the heterogeneity of assessments is a constraint to meta-analysis; a universal approach (key academic and cognitive outcomes, such as reading speed, comprehension, memory, etc.) must be agreed upon.

On the policymaking front, governments and educational institutions have to embrace technology. To begin with, validated AI and neurotech tools should be explicitly integrated into special education frameworks as part of the tiered systems of support. Ministries of education may, for example, finance pilot schemes that install adaptive reading software or EEG remediation in schools, with careful testing. However, general implementation should be postponed until their effectiveness is regarded as successful. Policymakers can prioritize the construction of evaluation systems and criteria, such as certification systems in edtech that assess efficacy, safety, and equity (cf. UNESCO noting that AI in education will need to address the ability to effectively accommodate students with learning impairments or disabilities). The teacher training curricula must be revised in parallel: according to UNESCO reports, although the majority of countries already have technology policy on teacher preparation, this is only the case in two-thirds of low-income countries. To ensure that humane pedagogy is observed by educators, we suggest that modules on AI literacy and neurotechnology should be included in the professional development of educators so that they can critically use the tools and continue to adhere to humane pedagogy (UNICEF, 2024).

## Limitations

While the reviewed interventions are promising, most existing studies are small in scale, short in duration, and methodologically heterogeneous. Replication through larger, well-controlled randomized trials with extended follow-up is essential to determine whether these early effects translate into sustained neuroplastic and literacy gains. A further limitation of this review is that performance metrics across studies are not directly comparable. Reported accuracy, sensitivity, specificity, and AUC values are derived from different datasets, languages, participant age groups, and evaluation protocols. Achieving fair and meaningful comparisons will require standardized benchmarking on common datasets, which remains a critical gap in the field.

## Conclusion

With rapid advances in neuroscience, artificial intelligence, and educational practice, this narrative review synthesizes emerging evidence in the evolving landscape of dyslexia diagnosis and intervention. Technological developments—including AI-powered diagnostic models, neurofeedback systems, and immersive VR-based training—are reshaping how dyslexia is identified, explained, and addressed. These innovations increasingly move beyond traditional one-size-fits-all approaches, offering personalized, scalable, and neurocognitively informed interventions.

AI applications such as intelligent tutoring systems, handwriting and speech analysis models, and real-time assistive tools show promising potential to support early identification of dyslexia and improve reading-relevant outcomes. Likewise, neuroscience-based interventions, including EEG-guided neurofeedback and brain–computer interface technologies, have demonstrated encouraging short-term effects on reading fluency, working memory, and phonological processing, particularly when embedded in structured educational programs. Integrative technologies that combine these approaches—for example, AI paired with neurofeedback or cognitive training games—are yielding early but promising results, pointing toward the value of multimodal, interdisciplinary strategies.

Despite these advances, major equity gaps persist. Access to digital dyslexia resources remains uneven across regions, income levels, and language groups. Low- and middle-income countries often lack the infrastructure, trained personnel, and culturally adapted content required to implement these tools effectively. Even in high-resource contexts, marginalized learners may remain excluded due to digital divides and policy inconsistencies. Furthermore, language adaptability is a critical concern: models trained on high-resource languages may not generalize well to other orthographies and phonologies, raising issues of fairness and diagnostic validity.

The ethical landscape surrounding AI and neurotechnology in dyslexia care requires careful governance. Strong safeguards are needed to protect data privacy, prevent algorithmic bias, and ensure age-appropriate consent and neuroethical handling of brain data. These considerations are particularly crucial when technologies involve children or other vulnerable populations.

Finally, technology must augment, not replace, human interaction. Effective dyslexia support depends on teachers, parents, clinicians, and caregivers who provide the emotional, cognitive, and social scaffolding that technology alone cannot replicate. Policy frameworks should prioritize equitable access, robust regulation, and the integration of these innovations into inclusive education systems. By embedding AI and neurotechnological tools within human-centered, culturally responsive practices, their promise can be harnessed responsibly to improve early detection and intervention for dyslexia.

## Data Availability

The original contributions presented in the study are included in the article/supplementary material, further inquiries can be directed to the corresponding author.
